# Supplementary material for: Highly thermostable carboxylic acid reductases generated by ancestral sequence reconstruction
Source: Commun Biol. 2019 Nov 22;2:429. doi: 10.1038/s42003-019-0677-y (PMC6874671; doi:10.1038/s42003-019-0677-y)
Supplement: Supplementary file 1 — Supplementary Information [file 42003_2019_677_MOESM1_ESM.pdf]

Supplementary Figure 1

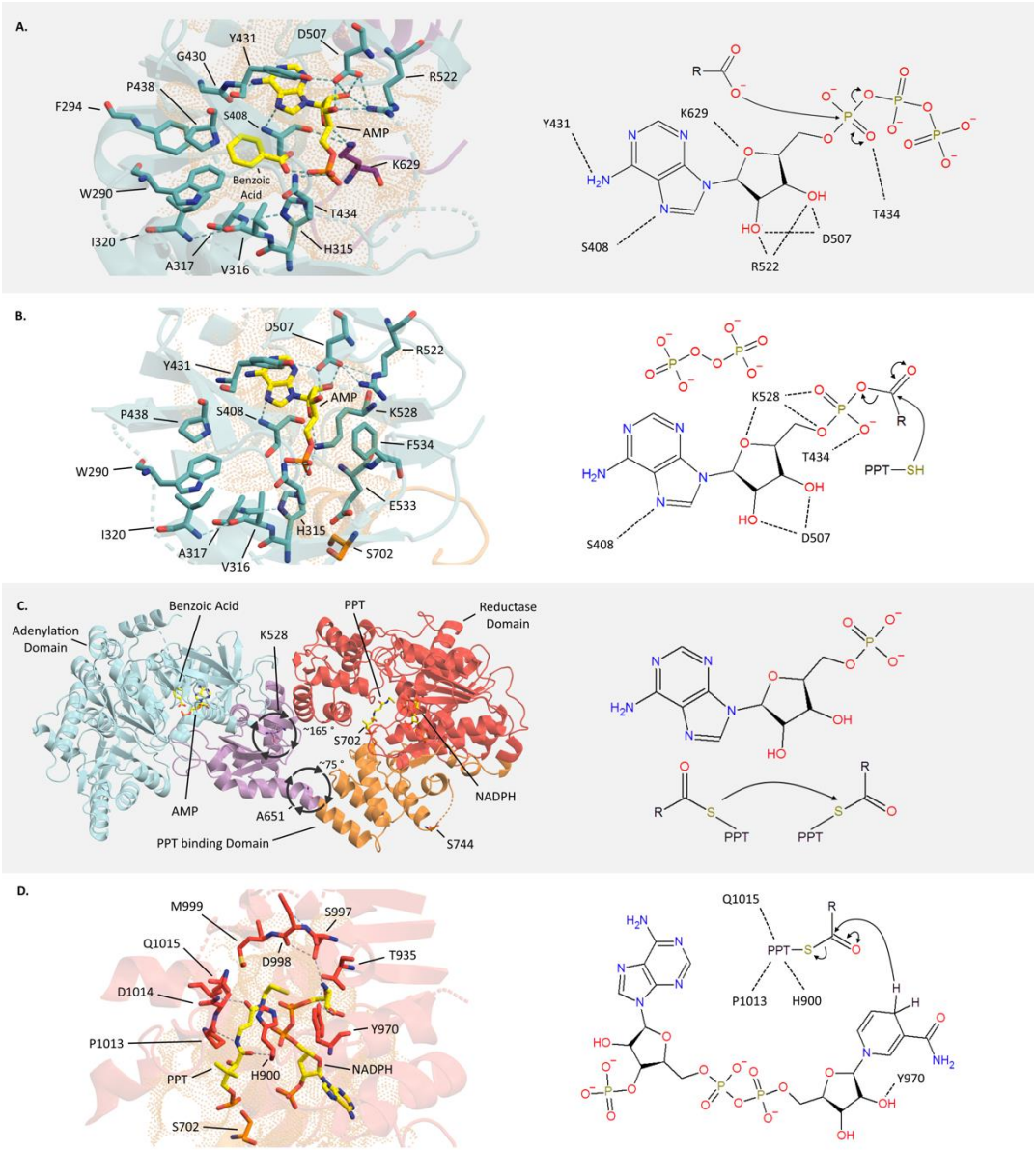

Supplementary Figure 1. Current proposed CAR reaction mechanism.

The current model of the reaction mechanism of CARs, based upon partial crystal structures of *Segnilliparus rusogus*, *Nocardia iowensis* and *Mycobacterium marinum* CARs [1]: **A.** ATP and carboxylic acid enter the adenylation domain active site leading to the formation of an acyl-AMP intermediate via nucleophilic attack of the carboxylate on the α-phosphate of ATP, releasing pyrophosphate. **B.** The adenylation domain is displaced due to rotation at residues L528 (~165°) and A651 (~75°), mediating the migration of the phosphopantetheine (PPT) arm (~50 Å; covalently bound to S702) into the adenylation domain active site. This movement permits nucleophilic attack by the PPT thiol on the carbonyl of the acyl-AMP intermediate, forming a thioester intermediate. **C.** The reductase domain undergoes conformational sampling about S744 and reconciles with the relocated PPT thioester intermediate. **D.** The thioester bond is reduced by NADPH releasing the aldehyde

product and $NADP^+$ and regenerating the PPT moiety (Crystal structures were rendered in PyMol v. 2.0; *PDB IDs: 5MSS, 5MST and, 5MSV*).

Supplementary figure 2

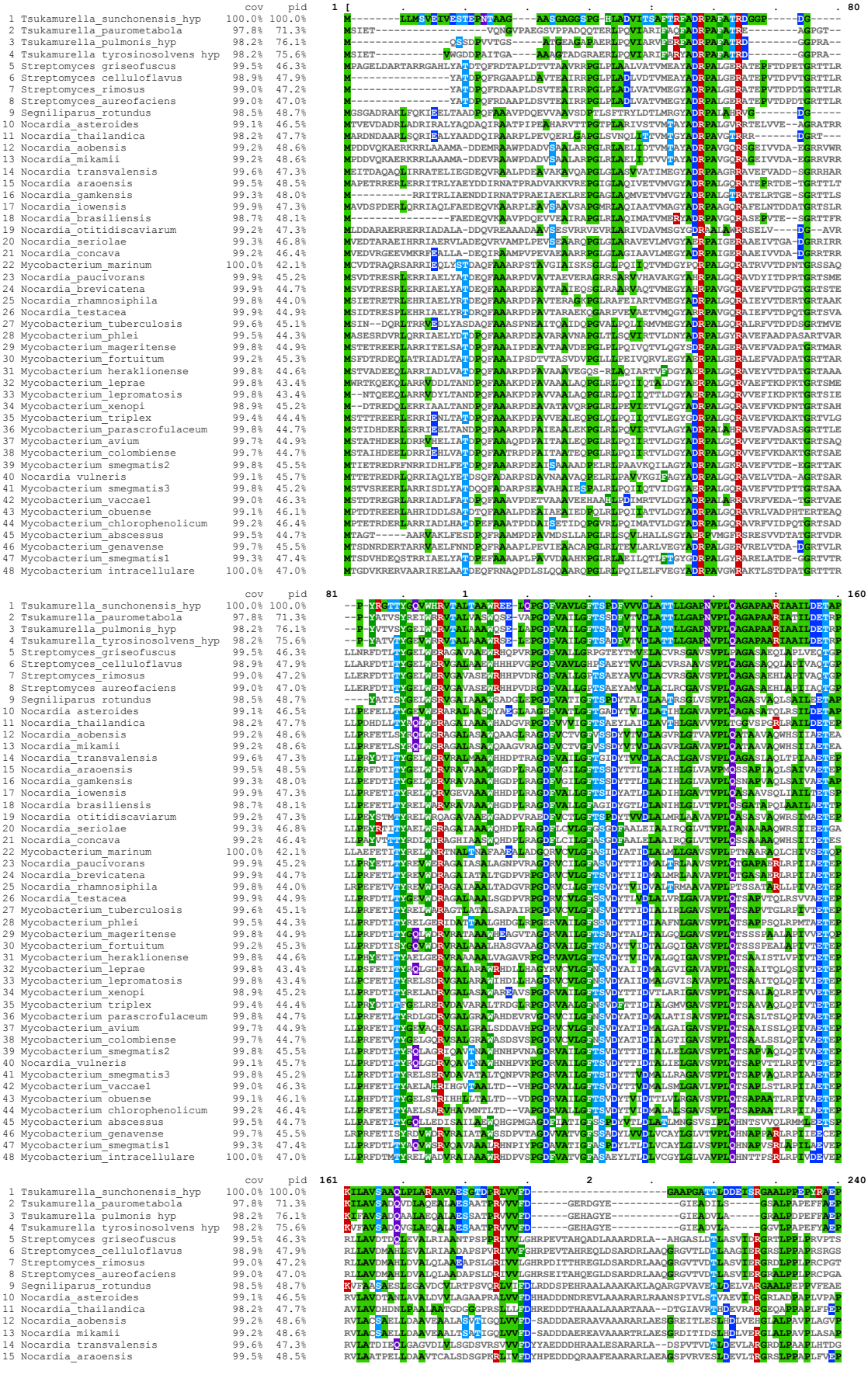

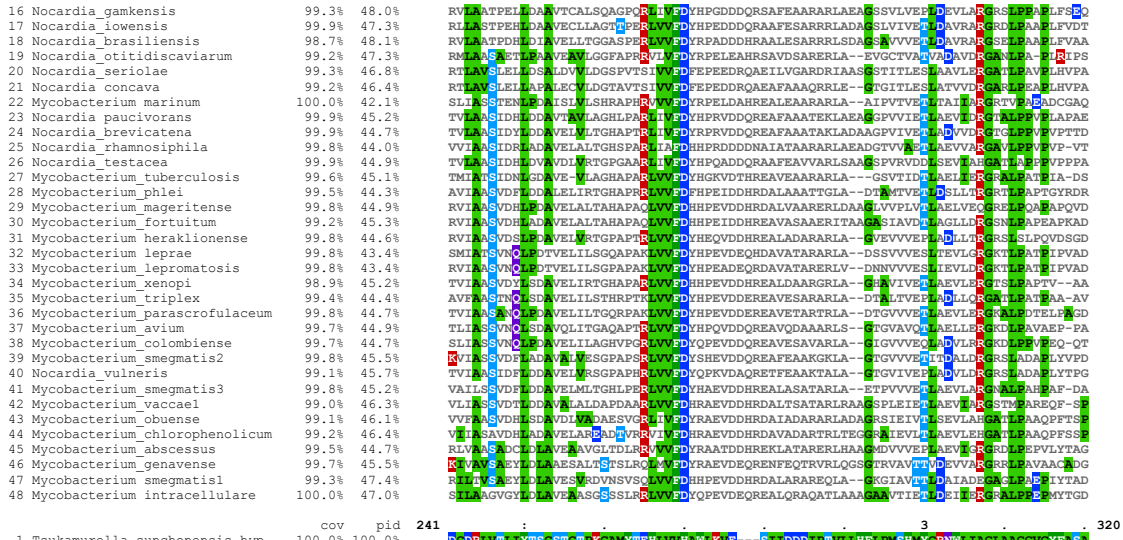
16 Nocardia_gamkensis 99.3% 48.0%
17 Nocardia_iowensis 99.9% 47.3%
18 Nocardia_brasiliensis 98.7% 48.1%
19 Nocardia_otitidiscaviarum 99.2% 47.3%
20 Nocardia_seriolae 99.3% 46.8%
21 Nocardia concava 99.2% 46.4%
22 Mycobacterium marinum 100.0% 42.1%
23 Nocardia paucivorans 99.9% 45.2%
24 Nocardia_brevicatena 99.9% 44.7%
25 Nocardia_rhamnosiphila 99.8% 44.0%
26 Nocardia_testacea 99.9% 44.9%
27 Mycobacterium_tuberculosis 99.6% 45.1%
28 Mycobacterium_phlei 99.5% 44.3%
29 Mycobacterium_mageritense 99.8% 44.9%
30 Mycobacterium_fortuitum 99.2% 45.3%
31 Mycobacterium heraklionense 99.8% 44.6%
32 Mycobacterium leprae 99.8% 43.4%
33 Mycobacterium_lepromatosis 99.8% 43.4%
34 Mycobacterium_xenopi 98.9% 45.2%
35 Mycobacterium_triplex 99.4% 44.4%
36 Mycobacterium_parascrofulaceum 99.8% 44.7%
37 Mycobacterium_avium 99.7% 44.9%
38 Mycobacterium_colombiense 99.7% 44.7%
39 Mycobacterium_smegmatis2 99.8% 45.5%
40 Nocardia_vulneris 99.1% 45.7%
41 Mycobacterium_smegmatis3 99.8% 45.2%
42 Mycobacterium_vaccae1 99.0% 46.3%
43 Mycobacterium_obuense 99.1% 46.1%
44 Mycobacterium_chlorophenolicum 99.2% 46.4%
45 Mycobacterium_abscessus 99.5% 44.7%
46 Mycobacterium_genavense 99.7% 45.5%
47 Mycobacterium_smegmatis1 99.3% 47.4%
48 Mycobacterium_intracellulare 100.0% 47.0%

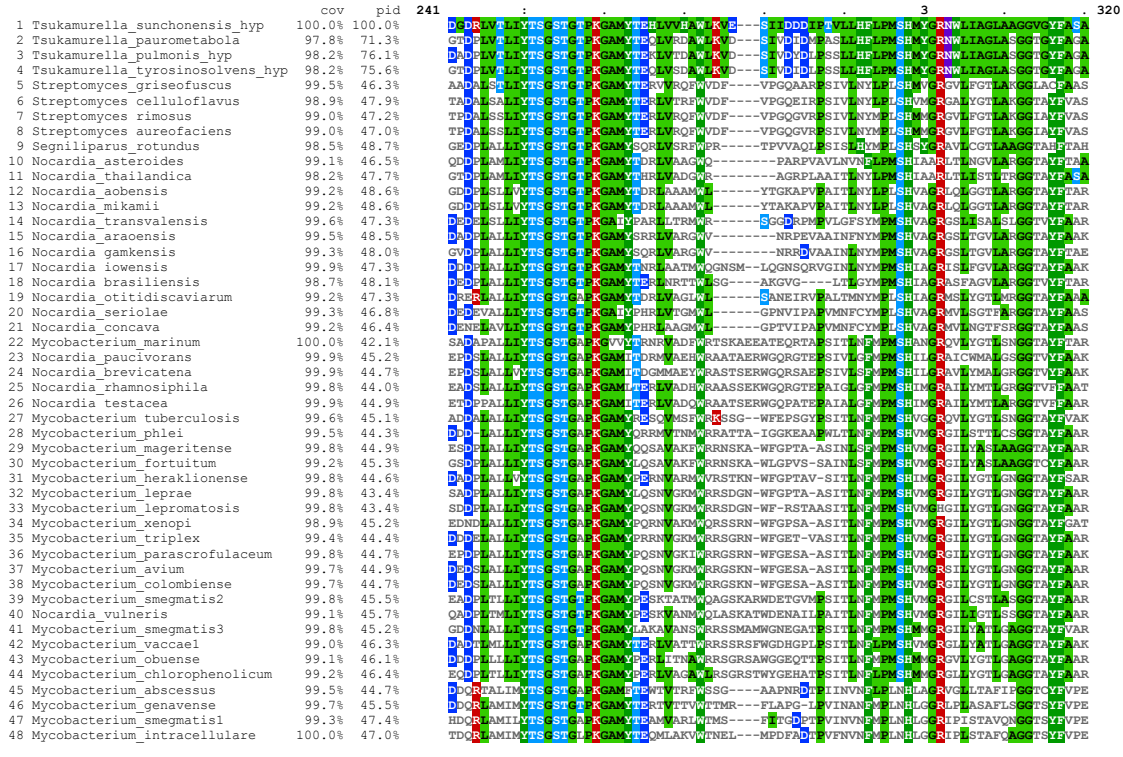
cov pid
1 Tsukamurella_sunchonensis_hyp 100.0% 100.0%
2 Tsukamurella_paurometabola 97.8% 71.3%
3 Tsukamurella_pulmonis_hyp 98.2% 76.1%
4 Tsukamurella_tyrosinosolvens_hyp 98.2% 75.6%
5 Streptomyces_griseofuscus 99.5% 46.3%
6 Streptomyces_celluloflavus 98.9% 47.9%
7 Streptomyces_rimosus 99.0% 47.2%
8 Streptomyces_aureofaciens 99.0% 47.0%
9 Segniliparus_rotundus 98.5% 48.7%
10 Nocardia_asteroides 99.1% 46.5%
11 Nocardia_thailandica 98.2% 47.7%
12 Nocardia_aobensis 99.2% 48.6%
13 Nocardia_mikamii 99.2% 48.6%
14 Nocardia_transvalensis 99.6% 47.3%
15 Nocardia_araoensis 99.5% 48.5%
16 Nocardia_gamkensis 99.3% 48.0%
17 Nocardia_iowensis 99.9% 47.3%
18 Nocardia_brasiliensis 98.7% 48.1%
19 Nocardia_otitidiscaviarum 99.2% 47.3%
20 Nocardia_seriolae 99.3% 46.8%
21 Nocardia_concava 99.2% 46.4%
22 Mycobacterium_marinum 100.0% 42.1%
23 Nocardia_paucivorans 99.9% 45.2%
24 Nocardia_brevicatena 99.9% 44.7%
25 Nocardia_rhamnosiphila 99.8% 44.0%
26 Nocardia_testacea 99.9% 44.9%
27 Mycobacterium_tuberculosis 99.6% 45.1%
28 Mycobacterium_phlei 99.5% 44.3%
29 Mycobacterium_mageritense 99.8% 44.9%
30 Mycobacterium_fortuitum 99.2% 45.3%
31 Mycobacterium_heraklionense 99.8% 44.6%
32 Mycobacterium_leprae 99.8% 43.4%
33 Mycobacterium_lepromatosis 99.8% 43.4%
34 Mycobacterium_xenopi 98.9% 45.2%
35 Mycobacterium_triplex 99.4% 44.4%
36 Mycobacterium_parascrofulaceum 99.8% 44.7%
37 Mycobacterium_avium 99.7% 44.9%
241
320

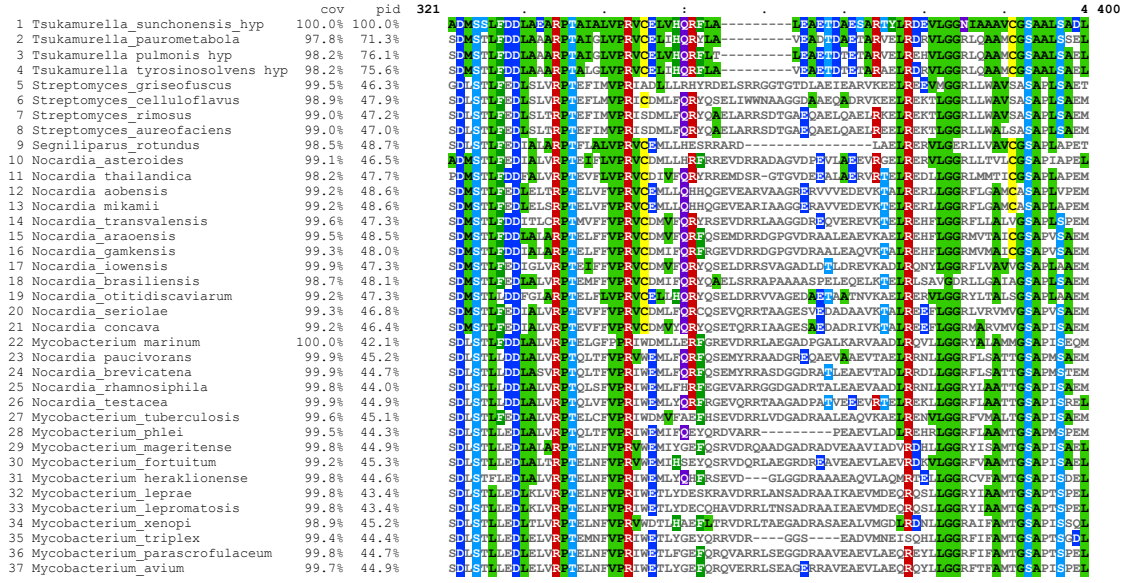
cov pid
1 Tsukamurella_sunchonensis_hyp 100.0% 100.0%
2 Tsukamurella_paurometabola 97.8% 71.3%
3 Tsukamurella_pulmonis_hyp 98.2% 76.1%
4 Tsukamurella_tyrosinosolvens_hyp 98.2% 75.6%
5 Streptomyces_griseofuscus 99.5% 46.3%
6 Streptomyces_celluloflavus 98.9% 47.9%
7 Streptomyces_rimosus 99.0% 47.2%
8 Streptomyces_aureofaciens 99.0% 47.0%
9 Segniliparus_rotundus 98.5% 48.7%
10 Nocardia_asteroides 99.1% 46.5%
11 Nocardia thailandica 98.2% 47.7%
12 Nocardia aobensis 99.2% 48.6%
13 Nocardia mikamii 99.2% 48.6%
14 Nocardia_transvalensis 99.6% 47.3%
15 Nocardia_araoensis 99.5% 48.5%
16 Nocardia_gamkensis 99.3% 48.0%
17 Nocardia_iowensis 99.9% 47.3%
18 Nocardia_brasiliensis 98.7% 48.1%
19 Nocardia_otitidiscaviarum 99.2% 47.3%
20 Nocardia_seriolae 99.3% 46.8%
21 Nocardia_concava 99.2% 46.4%
22 Mycobacterium marinum 100.0% 42.1%
23 Nocardia paucivorans 99.9% 45.2%
24 Nocardia_brevicatena 99.9% 44.7%
25 Nocardia_rhamnosiphila 99.8% 44.0%
26 Nocardia_testacea 99.9% 44.9%
27 Mycobacterium_tuberculosis 99.6% 45.1%
28 Mycobacterium_phlei 99.5% 44.3%
29 Mycobacterium_mageritense 99.8% 44.9%
30 Mycobacterium_fortuitum 99.2% 45.3%
31 Mycobacterium heraklionense 99.8% 44.6%
32 Mycobacterium_leprae 99.8% 43.4%
33 Mycobacterium_lepromatosis 99.8% 43.4%
34 Mycobacterium_xenopi 98.9% 45.2%
35 Mycobacterium_triplex 99.4% 44.4%
36 Mycobacterium_parascrofulaceum 99.8% 44.7%
37 Mycobacterium_avium 99.7% 44.9%
321
400

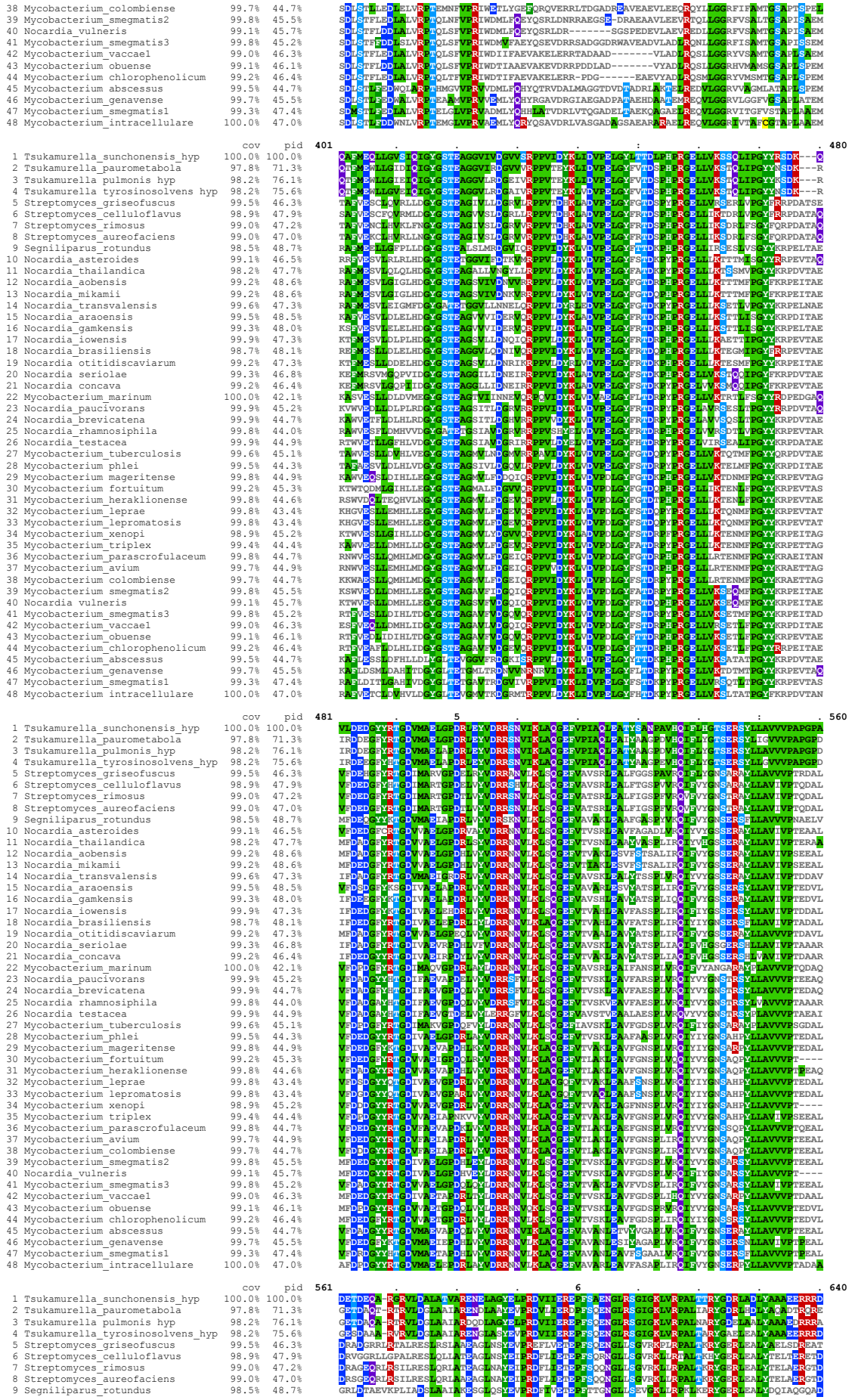
38 Mycobacterium_colombiense 99.7% 44.7%
39 Mycobacterium_smegmatis2 99.8% 45.5%
40 Nocardia_vulneris 99.1% 45.7%
41 Mycobacterium_smegmatis3 99.8% 45.2%
42 Mycobacterium_vaccae1 99.0% 46.3%
43 Mycobacterium obuense 99.1% 46.1%
44 Mycobacterium chlorophenolicum 99.2% 46.4%
45 Mycobacterium abscessus 99.5% 44.7%
46 Mycobacterium_genavense 99.7% 45.5%
47 Mycobacterium_smegmatis1 99.3% 47.4%
48 Mycobacterium_intracellulare 100.0% 47.0%
cov pid
1 Tsukamurella_sunchonensis_hyp 100.0% 100.0%
2 Tsukamurella_paurometabola 97.8% 71.3%
3 Tsukamurella pulmonis hyp 98.2% 76.1%
4 Tsukamurella tyrosinosolvens hyp 98.2% 75.6%
5 Streptomyces_griseofuscus 99.5% 46.3%
6 Streptomyces_celluloflavus 98.9% 47.9%
7 Streptomyces_rimosus 99.0% 47.2%
8 Streptomyces_aureofaciens 99.0% 47.0%
9 Segniliparus_rotundus 98.5% 48.7%
10 Nocardia_asteroides 99.1% 46.5%
11 Nocardia_thailandica 99.2% 47.7%
12 Nocardia_aobensis 99.2% 48.6%
13 Nocardia_mikamii 99.2% 48.6%
14 Nocardia_transvalensis 99.6% 47.3%
15 Nocardia_araoensis 99.5% 48.5%
16 Nocardia_gamkensis 99.3% 48.0%
17 Nocardia_iowensis 99.9% 47.3%
18 Nocardia_brasiliensis 98.7% 48.1%
19 Nocardia otitidiscaviarum 99.2% 47.3%
20 Nocardia seriolae 99.3% 46.8%
21 Nocardia concava 99.2% 46.4%
22 Mycobacterium_marinum 100.0% 42.1%
23 Nocardia_paucivorans 99.9% 45.2%
24 Nocardia_brevicatena 99.9% 44.7%
25 Nocardia_rhamnosiphila 99.8% 44.0%
26 Nocardia_testacea 99.9% 44.9%
27 Mycobacterium_tuberculosis 99.6% 45.1%
28 Mycobacterium_phlei 99.5% 44.3%
29 Mycobacterium_mageritense 99.8% 44.9%
30 Mycobacterium_fortuitum 99.2% 45.3%
31 Mycobacterium_heraklionense 99.8% 44.6%
32 Mycobacterium_leprae 99.8% 43.4%
33 Mycobacterium_lepromatosis 99.8% 43.4%
34 Mycobacterium xenopi 98.9% 45.2%
35 Mycobacterium triplex 99.4% 44.4%
36 Mycobacterium_parascrofulaceum 99.8% 44.7%
37 Mycobacterium_avium 99.7% 44.9%
38 Mycobacterium_colombiense 99.7% 44.7%
39 Mycobacterium_smegmatis2 99.8% 45.5%
40 Nocardia_vulneris 99.1% 45.7%
41 Mycobacterium_smegmatis3 99.8% 45.2%
42 Mycobacterium_vaccae1 99.0% 46.3%
43 Mycobacterium obuense 99.1% 46.1%
44 Mycobacterium chlorophenolicum 99.2% 46.4%
45 Mycobacterium abscessus 99.5% 44.7%
46 Mycobacterium_genavense 99.7% 45.5%
47 Mycobacterium_smegmatis1 99.3% 47.4%
48 Mycobacterium_intracellulare 100.0% 47.0%
cov pid
1 Tsukamurella_sunchonensis_hyp 100.0% 100.0%
2 Tsukamurella_paurometabola 97.8% 71.3%
3 Tsukamurella_pulmonis_hyp 98.2% 76.1%
4 Tsukamurella_tyrosinosolvens_hyp 98.2% 75.6%
5 Streptomyces_griseofuscus 99.5% 46.3%
6 Streptomyces_celluloflavus 98.9% 47.9%
7 Streptomyces_rimosus 99.0% 47.2%
8 Streptomyces_aureofaciens 99.0% 47.0%
9 Segniliparus_rotundus 98.5% 48.7%
10 Nocardia_asteroides 99.1% 46.5%
11 Nocardia_thailandica 99.2% 47.7%
12 Nocardia_aobensis 99.2% 48.6%
13 Nocardia_mikamii 99.2% 48.6%
14 Nocardia_transvalensis 99.6% 47.3%
15 Nocardia_araoensis 99.5% 48.5%
16 Nocardia_gamkensis 99.3% 48.0%
17 Nocardia_iowensis 99.9% 47.3%
18 Nocardia_brasiliensis 98.7% 48.1%
19 Nocardia_otitidiscaviarum 99.2% 47.3%
20 Nocardia_seriolae 99.3% 46.8%
21 Nocardia_concava 99.2% 46.4%
22 Mycobacterium_marinum 100.0% 42.1%
23 Nocardia_paucivorans 99.9% 45.2%
24 Nocardia_brevicatena 99.9% 44.7%
25 Nocardia rhamnosiphila 99.8% 44.0%
26 Nocardia testacea 99.9% 44.9%
27 Mycobacterium_tuberculosis 99.6% 45.1%
28 Mycobacterium_phlei 99.5% 44.3%
29 Mycobacterium_mageritense 99.8% 44.9%
30 Mycobacterium_fortuitum 99.2% 45.3%
31 Mycobacterium_heraklionense 99.8% 44.6%
32 Mycobacterium_leprae 99.8% 43.4%
33 Mycobacterium_lepromatosis 99.8% 43.4%
34 Mycobacterium xenopi 98.9% 45.2%
35 Mycobacterium triplex 99.4% 44.4%
36 Mycobacterium_parascrofulaceum 99.8% 44.7%
37 Mycobacterium_avium 99.7% 44.9%
38 Mycobacterium_colombiense 99.7% 44.7%
39 Mycobacterium_smegmatis2 99.8% 45.5%
40 Nocardia_vulneris 99.1% 45.7%
41 Mycobacterium_smegmatis3 99.8% 45.2%
42 Mycobacterium_vaccae1 99.0% 46.3%
43 Mycobacterium obuense 99.1% 46.1%
44 Mycobacterium chlorophenolicum 99.2% 46.4%
45 Mycobacterium abscessus 99.5% 44.7%
46 Mycobacterium_genavense 99.7% 45.5%
47 Mycobacterium_smegmatis1 99.3% 47.4%
48 Mycobacterium_intracellulare 100.0% 47.0%
cov pid
1 Tsukamurella_sunchonensis_hyp 100.0% 100.0%
2 Tsukamurella_paurometabola 97.8% 71.3%
3 Tsukamurella_pulmonis_hyp 98.2% 76.1%
4 Tsukamurella_tyrosinosolvens_hyp 98.2% 75.6%
5 Streptomyces_griseofuscus 99.5% 46.3%
6 Streptomyces_celluloflavus 98.9% 47.9%
7 Streptomyces_rimosus 99.0% 47.2%
8 Streptomyces_aureofaciens 99.0% 47.0%
9 Segniliparus_rotundus 98.5% 48.7%

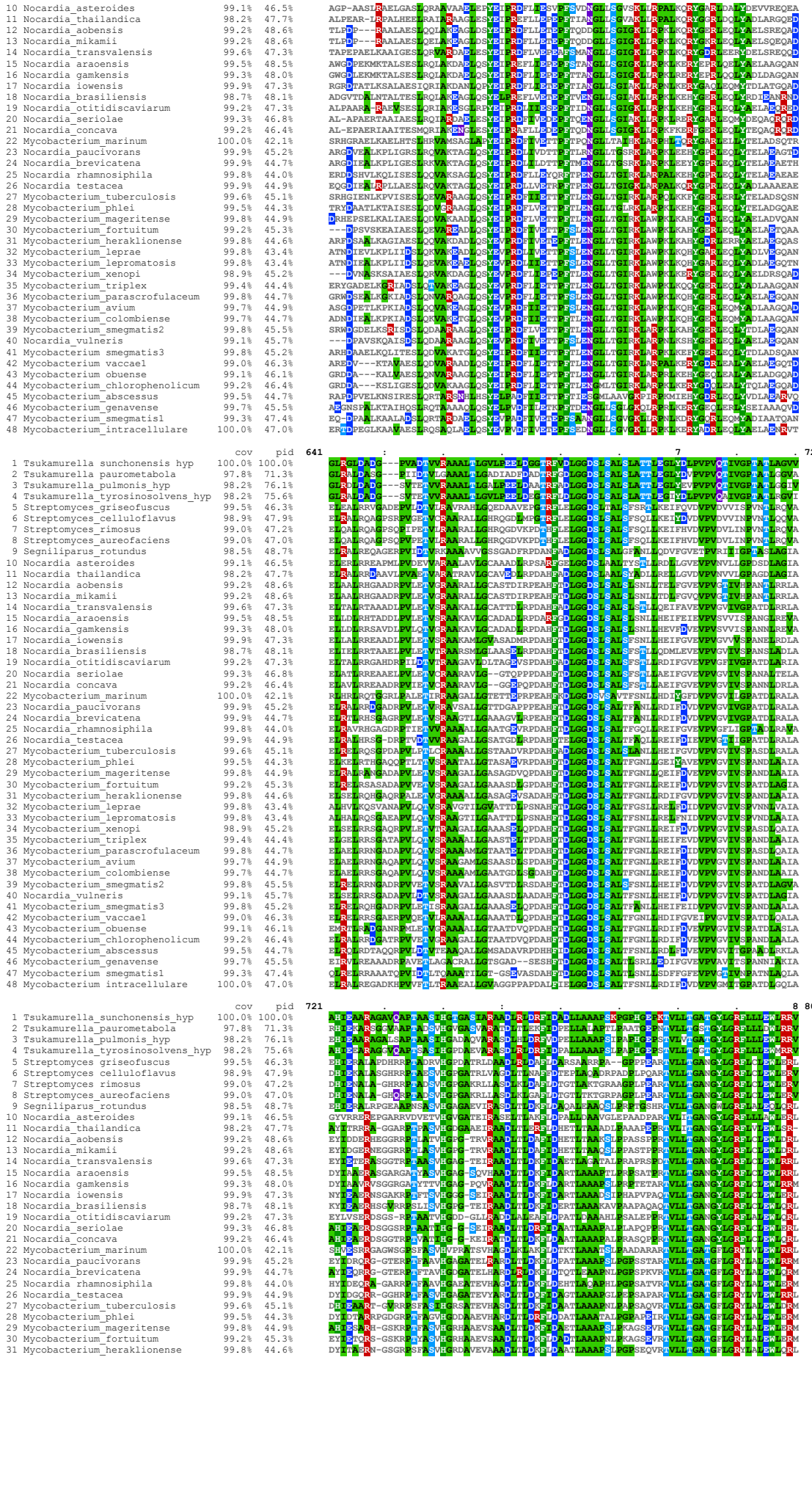
10 Nocardia_asteroides 99.1% 46.5%
11 Nocardia_thailandica 98.2% 47.7%
12 Nocardia_aobensis 99.2% 48.6%
13 Nocardia_mikamii 99.2% 48.6%
14 Nocardia_transvalensis 99.6% 47.3%
15 Nocardia araoensis 99.5% 48.5%
16 Nocardia gamkensis 99.3% 48.0%
17 Nocardia iowensis 99.9% 47.3%
18 Nocardia_brasiliensis 98.7% 48.1%
19 Nocardia_otitidiscaviarum 99.2% 47.3%
20 Nocardia_seriolae 99.3% 46.8%
21 Nocardia_concava 99.2% 46.4%
22 Mycobacterium_marinum 100.0% 42.1%
23 Nocardia_paucivorans 99.9% 45.2%
24 Nocardia_brevicatena 99.9% 44.7%
25 Nocardia rhamnosiphila 99.8% 44.0%
26 Nocardia testacea 99.9% 44.9%
27 Mycobacterium_tuberculosis 99.6% 45.1%
28 Mycobacterium_phlei 99.5% 44.3%
29 Mycobacterium_mageritense 99.8% 44.9%
30 Mycobacterium_fortuitum 99.2% 45.3%
31 Mycobacterium_heraklionense 99.8% 44.6%
32 Mycobacterium_leprae 99.8% 43.4%
33 Mycobacterium_lepromatosis 99.8% 43.4%
34 Mycobacterium_xenopi 98.9% 45.2%
35 Mycobacterium_triplex 99.4% 44.4%
36 Mycobacterium_parascrofulaceum 99.8% 44.7%
37 Mycobacterium_avium 99.7% 44.9%
38 Mycobacterium_colombiense 99.7% 44.7%
39 Mycobacterium_smegmatis2 99.8% 45.5%
40 Nocardia_vulneris 99.1% 45.7%
41 Mycobacterium smegmatis3 99.8% 45.2%
42 Mycobacterium vaccae1 99.0% 46.3%
43 Mycobacterium obuense 99.1% 46.1%
44 Mycobacterium_chlorophenolicum 99.2% 46.4%
45 Mycobacterium_abscessus 99.5% 44.7%
46 Mycobacterium_genavense 99.7% 45.5%
47 Mycobacterium_smegmatis1 99.3% 47.4%
48 Mycobacterium_intracellulare 100.0% 47.0%
cov pid
641
7
720
1 Tsukamurella sunchonensis hyp 100.0% 100.0%
2 Tsukamurella paurometabola 97.8% 71.3%
3 Tsukamurella_pulmonis_hyp 98.2% 76.1%
4 Tsukamurella_tyrosinosolvens_hyp 98.2% 75.6%
5 Streptomyces_griseofuscus 99.5% 46.3%
6 Streptomyces_celluloflavus 98.9% 47.9%
7 Streptomyces_rimosus 99.0% 47.2%
8 Streptomyces_aureofaciens 99.0% 47.0%
9 Segniliparus_rotundus 98.5% 48.7%
10 Nocardia_asteroides 99.1% 46.5%
11 Nocardia_thailandica 98.2% 47.7%
12 Nocardia_aobensis 99.2% 48.6%
13 Nocardia_mikamii 99.2% 48.6%
14 Nocardia_transvalensis 99.6% 47.3%
15 Nocardia_araoensis 99.5% 48.5%
16 Nocardia_gamkensis 99.3% 48.0%
17 Nocardia_iowensis 99.9% 47.3%
18 Nocardia_brasiliensis 98.7% 48.1%
19 Nocardia_otitidiscaviarum 99.2% 47.3%
20 Nocardia_seriolae 99.3% 46.8%
21 Nocardia_concava 99.2% 46.4%
22 Mycobacterium_marinum 100.0% 42.1%
23 Nocardia_paucivorans 99.9% 45.2%
24 Nocardia_brevicatena 99.9% 44.7%
25 Nocardia_rhamnosiphila 99.8% 44.0%
26 Nocardia_testacea 99.9% 44.9%
27 Mycobacterium_tuberculosis 99.6% 45.1%
28 Mycobacterium_phlei 99.5% 44.3%
29 Mycobacterium_mageritense 99.8% 44.9%
30 Mycobacterium_fortuitum 99.2% 45.3%
31 Mycobacterium_heraklionense 99.8% 44.6%
721
8
800

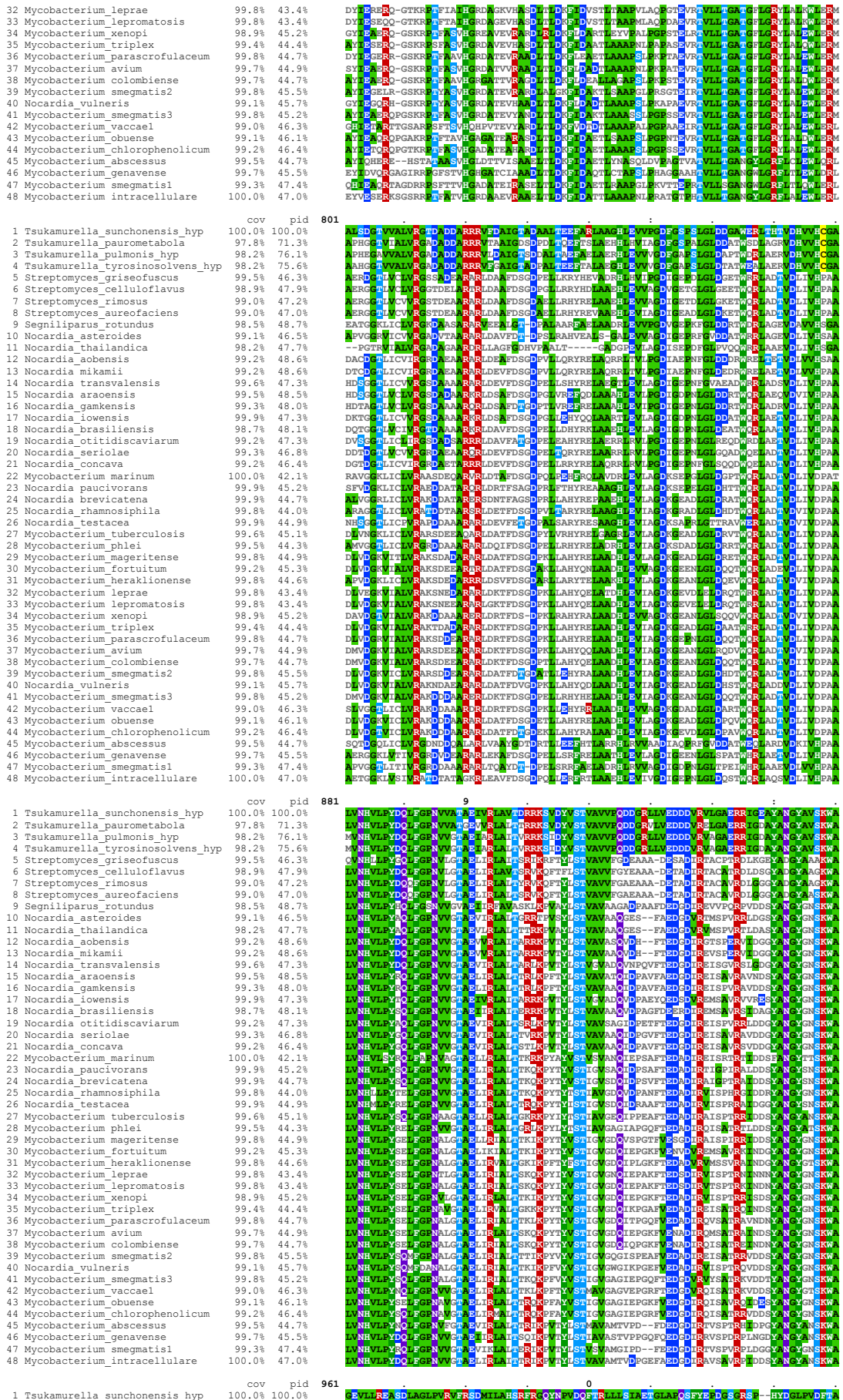
cov pid 801 880
1 Tsukamurella_sunchonensis_hyp 100.0% 100.0%
2 Tsukamurella_paurometabola 97.8% 71.3%
3 Tsukamurella_pulmonis_hyp 98.2% 76.1%
4 Tsukamurella_tyrosinosolvens_hyp 98.2% 75.6%
5 Streptomyces_griseofuscus 99.5% 46.3%
6 Streptomyces_celluloflavus 98.9% 47.9%
7 Streptomyces_rimosus 99.0% 47.2%
8 Streptomyces_aureofaciens 99.0% 47.0%
9 Segniliparus_rotundus 98.5% 48.7%
10 Nocardia_asteroides 99.1% 46.5%
11 Nocardia_thailandica 98.2% 47.7%
12 Nocardia_aobensis 99.2% 48.6%
13 Nocardia_mikamii 99.2% 48.6%
14 Nocardia_transvalensis 99.6% 47.3%
15 Nocardia_araoensis 99.5% 48.5%
16 Nocardia_gamkensis 99.3% 48.0%
17 Nocardia_iowensis 99.9% 47.3%
18 Nocardia_brasiliensis 98.7% 48.1%
19 Nocardia_otitidiscaviarum 99.2% 47.3%
20 Nocardia_seriolae 99.3% 46.8%
21 Nocardia_concava 99.2% 46.4%
22 Mycobacterium_marinum 100.0% 42.1%
23 Nocardia_paucivorans 99.9% 45.2%
24 Nocardia_brevicatena 99.9% 44.7%
25 Nocardia_rhamnosiphila 99.8% 44.0%
26 Nocardia_testacea 99.9% 44.9%
27 Mycobacterium_tuberculosis 99.6% 45.1%
28 Mycobacterium_phlei 99.5% 44.3%
29 Mycobacterium_mageritense 99.8% 44.9%
30 Mycobacterium_fortuitum 99.2% 45.3%
31 Mycobacterium_heraklionense 99.8% 44.6%
32 Mycobacterium_leprae 99.8% 43.4%
33 Mycobacterium_lepromatosis 99.8% 43.4%
34 Mycobacterium_xenopi 98.9% 45.2%
35 Mycobacterium_triplex 99.4% 44.4%
36 Mycobacterium_parascrofulaceum 99.8% 44.7%
37 Mycobacterium_avium 99.7% 44.9%
38 Mycobacterium_colombiense 99.7% 44.7%
39 Mycobacterium_smegmatis2 99.8% 45.5%
40 Nocardia_vulneris 99.1% 45.7%
41 Mycobacterium_smegmatis3 99.8% 45.2%
42 Mycobacterium_vaccae1 99.0% 46.3%
43 Mycobacterium_obuense 99.1% 46.1%
44 Mycobacterium_chlorophenolicum 99.2% 46.4%
45 Mycobacterium_abscessus 99.5% 44.7%
46 Mycobacterium_genavense 99.7% 45.5%
47 Mycobacterium_smegmatis1 99.3% 47.4%
48 Mycobacterium_intracellulare 100.0% 47.0%
cov pid 881 960
1 Tsukamurella_sunchonensis_hyp 100.0% 100.0%
2 Tsukamurella_paurometabola 97.8% 71.3%
3 Tsukamurella_pulmonis_hyp 98.2% 76.1%
4 Tsukamurella_tyrosinosolvens_hyp 98.2% 75.6%
5 Streptomyces_griseofuscus 99.5% 46.3%
6 Streptomyces_celluloflavus 98.9% 47.9%
7 Streptomyces_rimosus 99.0% 47.2%
8 Streptomyces_aureofaciens 99.0% 47.0%
9 Segniliparus_rotundus 98.5% 48.7%
10 Nocardia_asteroides 99.1% 46.5%
11 Nocardia_thailandica 98.2% 47.7%
12 Nocardia_aobensis 99.2% 48.6%
13 Nocardia_mikamii 99.2% 48.6%
14 Nocardia_transvalensis 99.6% 47.3%
15 Nocardia_araoensis 99.5% 48.5%
16 Nocardia_gamkensis 99.3% 48.0%
17 Nocardia_iowensis 99.9% 47.3%
18 Nocardia_brasiliensis 98.7% 48.1%
19 Nocardia_otitidiscaviarum 99.2% 47.3%
20 Nocardia_seriolae 99.3% 46.8%
21 Nocardia_concava 99.2% 46.4%
22 Mycobacterium_marinum 100.0% 42.1%
23 Nocardia_paucivorans 99.9% 45.2%
24 Nocardia_brevicatena 99.9% 44.7%
25 Nocardia_rhamnosiphila 99.8% 44.0%
26 Nocardia_testacea 99.9% 44.9%
27 Mycobacterium_tuberculosis 99.6% 45.1%
28 Mycobacterium_phlei 99.5% 44.3%
29 Mycobacterium_mageritense 99.8% 44.9%
30 Mycobacterium_fortuitum 99.2% 45.3%
31 Mycobacterium_heraklionense 99.8% 44.6%
32 Mycobacterium_leprae 99.8% 43.4%
33 Mycobacterium_lepromatosis 99.8% 43.4%
34 Mycobacterium_xenopi 98.9% 45.2%
35 Mycobacterium_triplex 99.4% 44.4%
36 Mycobacterium_parascrofulaceum 99.8% 44.7%
37 Mycobacterium_avium 99.7% 44.9%
38 Mycobacterium_colombiense 99.7% 44.7%
39 Mycobacterium_smegmatis2 99.8% 45.5%
40 Nocardia_vulneris 99.1% 45.7%
41 Mycobacterium_smegmatis3 99.8% 45.2%
42 Mycobacterium_vaccae1 99.0% 46.3%
43 Mycobacterium_obuense 99.1% 46.1%
44 Mycobacterium_chlorophenolicum 99.2% 46.4%
45 Mycobacterium_abscessus 99.5% 44.7%
46 Mycobacterium_genavense 99.7% 45.5%
47 Mycobacterium_smegmatis1 99.3% 47.4%
48 Mycobacterium_intracellulare 100.0% 47.0%
cov pid 961 1040
1 Tsukamurella_sunchonensis_hyp 100.0% 100.0%
2 Tsukamurella_paurometabola 97.8% 71.3%
3 Tsukamurella_pulmonis_hyp 98.2% 76.1%

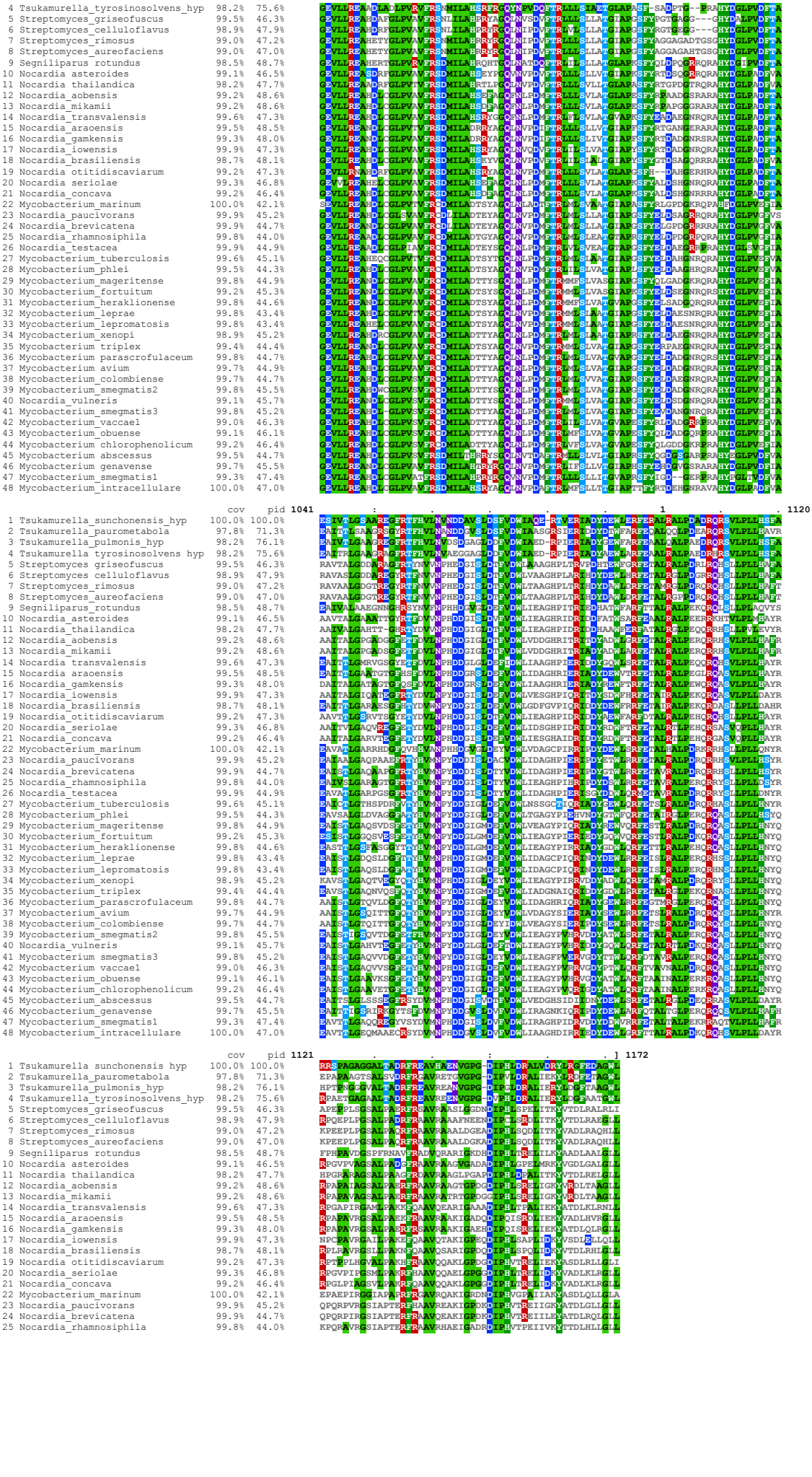
4 Tsukamurella_tyrosinosolvens_hyp 98.2% 75.6%
5 Streptomyces_griseofuscus 99.5% 46.3%
6 Streptomyces_celluloflavus 98.9% 47.9%
7 Streptomyces_rimosus 99.0% 47.2%
8 Streptomyces_aureofaciens 99.0% 47.0%
9 Segniliparus_rotundus 98.5% 48.7%
10 Nocardia_asteroides 99.1% 46.5%
11 Nocardia_thailandica 98.2% 47.7%
12 Nocardia_aobensis 99.2% 48.6%
13 Nocardia_mikamii 99.2% 48.6%
14 Nocardia_transvalensis 99.6% 47.3%
15 Nocardia_araoensis 99.5% 48.5%
16 Nocardia_gamkensis 99.3% 48.0%
17 Nocardia_iowensis 99.9% 47.3%
18 Nocardia_brasiliensis 98.7% 48.1%
19 Nocardia_otitidiscaviarum 99.2% 47.3%
20 Nocardia_seriolae 99.3% 46.8%
21 Nocardia_concava 99.2% 46.4%
22 Mycobacterium_marinum 100.0% 42.1%
23 Nocardia_paucivorans 99.9% 45.2%
24 Nocardia_brevicatena 99.9% 44.7%
25 Nocardia_rhamnosiphila 99.8% 44.0%
26 Nocardia_testacea 99.9% 44.9%
27 Mycobacterium_tuberculosis 99.6% 45.1%
28 Mycobacterium_phlei 99.5% 44.3%
29 Mycobacterium_mageritense 99.8% 44.9%
30 Mycobacterium_fortuitum 99.2% 45.3%
31 Mycobacterium_heraklionense 99.8% 44.6%
32 Mycobacterium_leprae 99.8% 43.4%
33 Mycobacterium_lepromatosis 99.8% 43.4%
34 Mycobacterium_xenopi 98.9% 45.2%
35 Mycobacterium triplex 99.4% 44.4%
36 Mycobacterium parascrofulaceum 99.8% 44.7%
37 Mycobacterium avium 99.7% 44.9%
38 Mycobacterium_colombiense 99.7% 44.7%
39 Mycobacterium_smegmatis2 99.8% 45.5%
40 Nocardia_vulneris 99.1% 45.7%
41 Mycobacterium_smegmatis3 99.8% 45.2%
42 Mycobacterium_vaccae1 99.0% 46.3%
43 Mycobacterium_obuense 99.1% 46.1%
44 Mycobacterium_chlorophenolicum 99.2% 46.4%
45 Mycobacterium_abscessus 99.5% 44.7%
46 Mycobacterium_genavense 99.7% 45.5%
47 Mycobacterium_smegmatis1 99.3% 47.4%
48 Mycobacterium_intracellulare 100.0% 47.0%
cov
pid
1041
1
1120
1 Tsukamurella_sunchonensis_hyp 100.0% 100.0%
2 Tsukamurella_paurometabola 97.8% 71.3%
3 Tsukamurella_pulmonis_hyp 98.2% 76.1%
4 Tsukamurella tyrosinosolvens hyp 98.2% 75.6%
5 Streptomyces griseofuscus 99.5% 46.3%
6 Streptomyces celluloflavus 98.9% 47.9%
7 Streptomyces_rimosus 99.0% 47.2%
8 Streptomyces_aureofaciens 99.0% 47.0%
9 Segniliparus_rotundus 98.5% 48.7%
10 Nocardia_asteroides 99.1% 46.5%
11 Nocardia_thailandica 98.2% 47.7%
12 Nocardia_aobensis 99.2% 48.6%
13 Nocardia_mikamii 99.2% 48.6%
14 Nocardia transvalensis 99.6% 47.3%
15 Nocardia araoensis 99.5% 48.5%
16 Nocardia_gamkensis 99.3% 48.0%
17 Nocardia_iowensis 99.9% 47.3%
18 Nocardia_brasiliensis 98.7% 48.1%
19 Nocardia_otitidiscaviarum 99.2% 47.3%
20 Nocardia_seriolae 99.3% 46.8%
21 Nocardia_concava 99.2% 46.4%
22 Mycobacterium_marinum 100.0% 42.1%
23 Nocardia_paucivorans 99.9% 45.2%
24 Nocardia_brevicatena 99.9% 44.7%
25 Nocardia_rhamnosiphila 99.8% 44.0%
26 Nocardia_testacea 99.9% 44.9%
27 Mycobacterium_tuberculosis 99.6% 45.1%
28 Mycobacterium_phlei 99.5% 44.3%
29 Mycobacterium_mageritense 99.8% 44.9%
30 Mycobacterium_fortuitum 99.2% 45.3%
31 Mycobacterium_heraklionense 99.8% 44.6%
32 Mycobacterium_leprae 99.8% 43.4%
33 Mycobacterium_lepromatosis 99.8% 43.4%
34 Mycobacterium_xenopi 98.9% 45.2%
35 Mycobacterium_triplex 99.4% 44.4%
36 Mycobacterium_parascrofulaceum 99.8% 44.7%
37 Mycobacterium_avium 99.7% 44.9%
38 Mycobacterium_colombiense 99.7% 44.7%
39 Mycobacterium_smegmatis2 99.8% 45.5%
40 Nocardia_vulneris 99.1% 45.7%
41 Mycobacterium smegmatis3 99.8% 45.2%
42 Mycobacterium_vaccae1 99.0% 46.3%
43 Mycobacterium_obuense 99.1% 46.1%
44 Mycobacterium_chlorophenolicum 99.2% 46.4%
45 Mycobacterium_abscessus 99.5% 44.7%
46 Mycobacterium_genavense 99.7% 45.5%
47 Mycobacterium_smegmatis1 99.3% 47.4%
48 Mycobacterium_intracellulare 100.0% 47.0%
cov
pid
1121
1172
1 Tsukamurella sunchonensis hyp 100.0% 100.0%
2 Tsukamurella_paurometabola 97.8% 71.3%
3 Tsukamurella_pulmonis_hyp 98.2% 76.1%
4 Tsukamurella_tyrosinosolvens_hyp 98.2% 75.6%
5 Streptomyces_griseofuscus 99.5% 46.3%
6 Streptomyces_celluloflavus 98.9% 47.9%
7 Streptomyces_rimosus 99.0% 47.2%
8 Streptomyces_aureofaciens 99.0% 47.0%
9 Segniliparus_rotundus 98.5% 48.7%
10 Nocardia asteroides 99.1% 46.5%
11 Nocardia thailandica 98.2% 47.7%
12 Nocardia_aobensis 99.2% 48.6%
13 Nocardia_mikamii 99.2% 48.6%
14 Nocardia_transvalensis 99.6% 47.3%
15 Nocardia_araoensis 99.5% 48.5%
16 Nocardia_gamkensis 99.3% 48.0%
17 Nocardia_iowensis 99.9% 47.3%
18 Nocardia_brasiliensis 98.7% 48.1%
19 Nocardia_otitidiscaviarum 99.2% 47.3%
20 Nocardia_seriolae 99.3% 46.8%
21 Nocardia_concava 99.2% 46.4%
22 Mycobacterium_marinum 100.0% 42.1%
23 Nocardia_paucivorans 99.9% 45.2%
24 Nocardia_brevicatena 99.9% 44.7%
25 Nocardia_rhamnosiphila 99.8% 44.0%

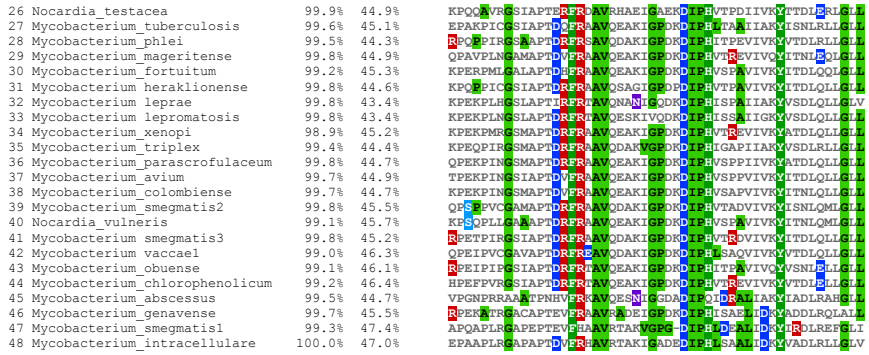

Supplementary figure 2 – CAR alignment used for reconstruction

CARs sequences were selected from dataset of 124 CAR sequences we previously published [2] to broadly cover sequence space and include a number of previously analysed CAR enzymes. CARs were aligned in MUSCLE and cured of noisy sites by eye within the Geneious software suite.

Supplementary figure 3

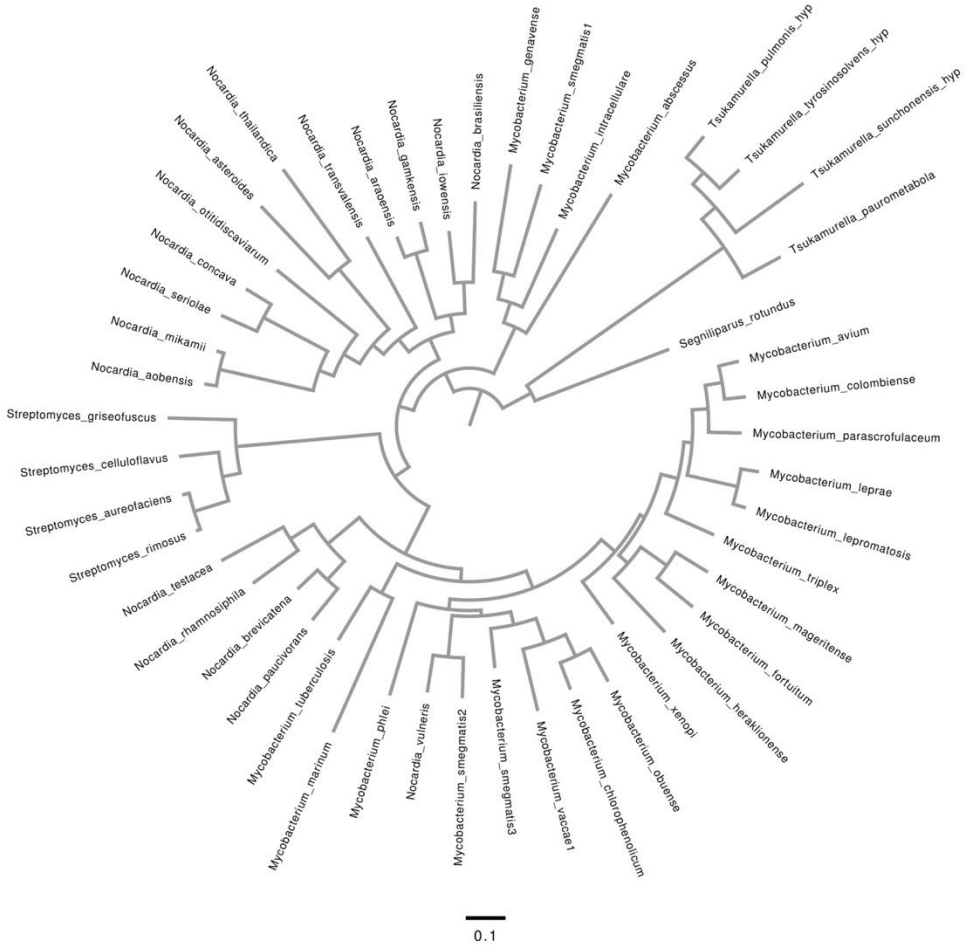

Supplementary Figure 3 – CAR phylogeny with leaf names

Equivalent phylogeny to Figure 2 with leaf names. Phylogeny was constructed in MrBayes [3] under the, WAG+I+G model of amino acid substitution [4], with *Tsukamurella* constrained to the outgroup and independent gamma rates being sampled across all lineages to account for rate homogeneity. Tree was configured in FigTree v. 1.4.3 and edited with Gravit designer. The Scale-bar represents amino acid changes per site.

Supplementary figure 4

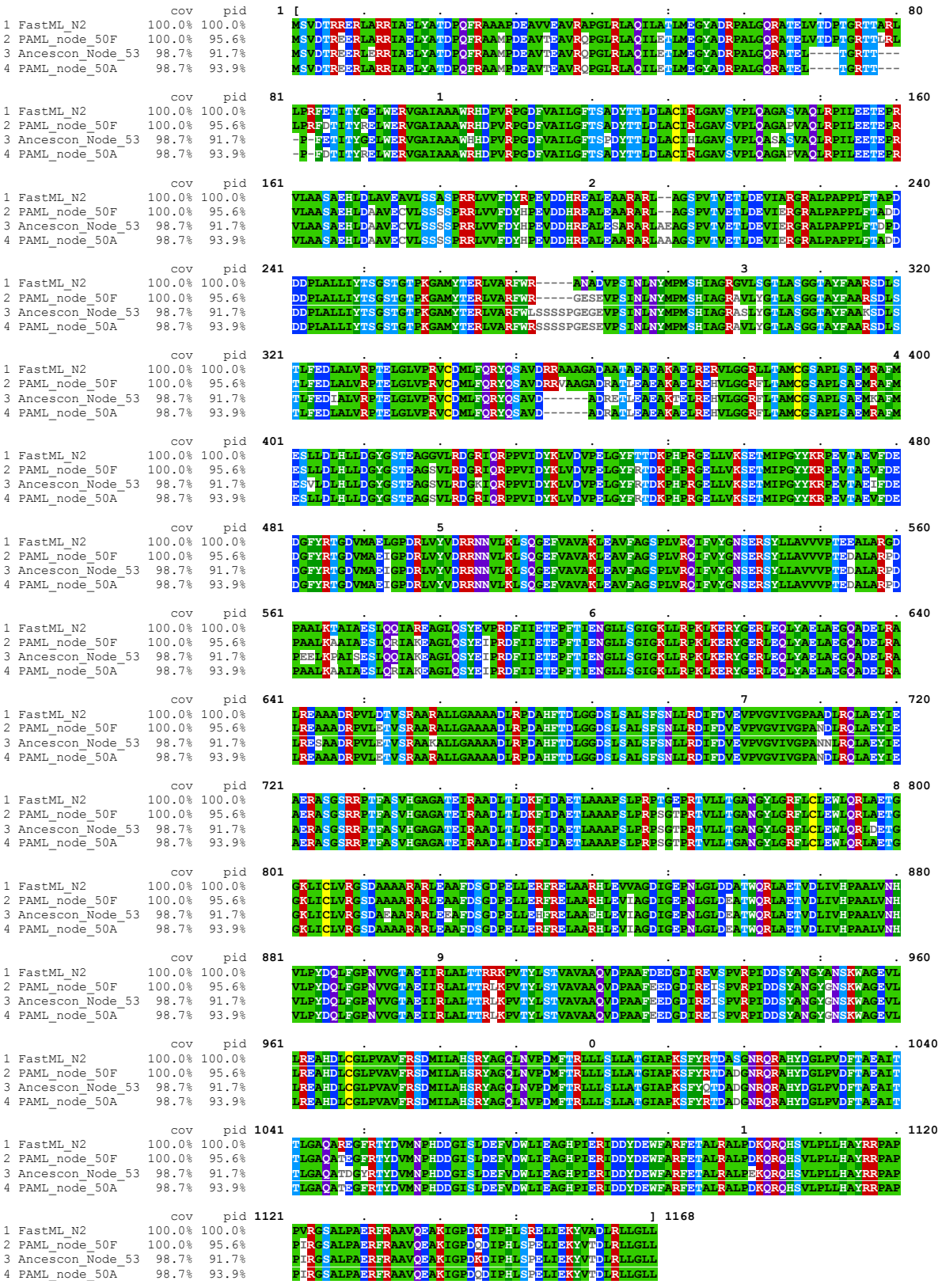

Supplementary Figure 4 - Alignment of AncCAR protein sequences

AncCAR sequences were produced with the Ancescon, PAML and FastML algorithms from the tree in Figure 2A and the alignment in Supplementary figure 1. The most likely sequence from the posterior probability distribution of the most ancestral node were taken as the ancestral state for each algorithm. Sequences were aligned with MUSCLE in the Geneious v. 10.0 software suite, and visualised using MVIEW v.1.63 (https://www.ebi.ac.uk/Tools/msa/mview/) [5].

Supplementary Figure 5

A

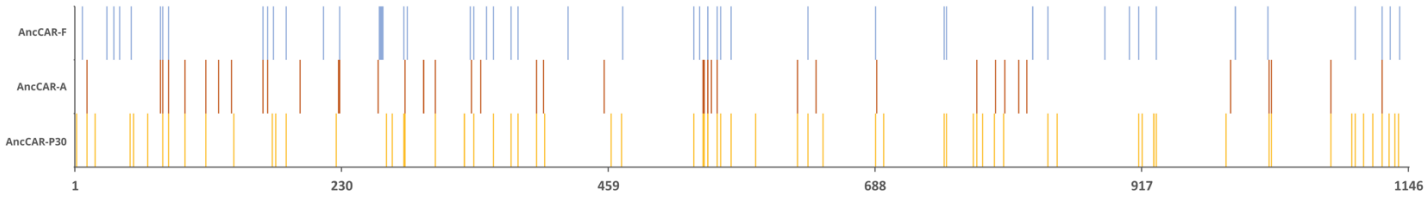

B

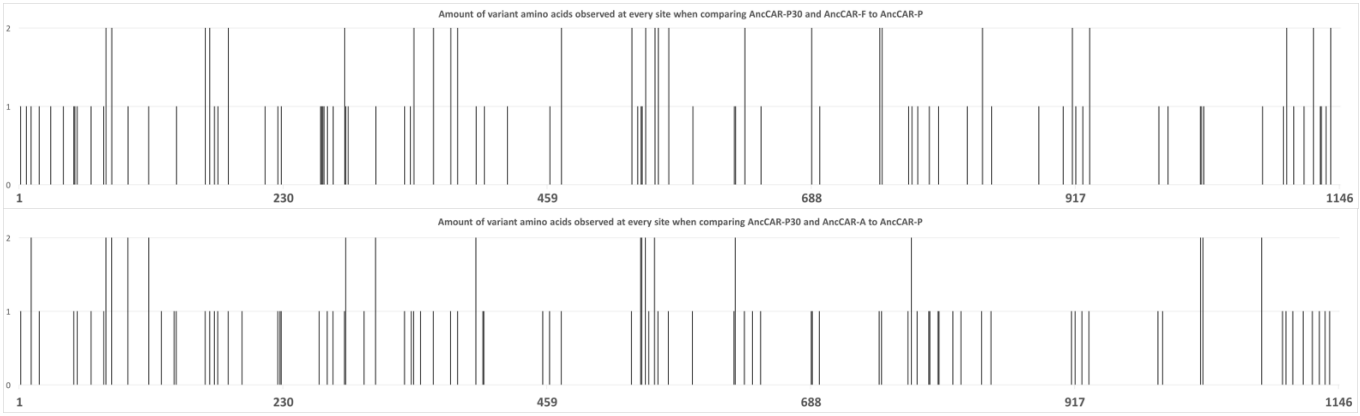

	Number of residues that vary in the dataset compared to AncCAR-P	Number of residues varying at a position in both algorithms compared to AncCAR-P	% residues with shared diversity compared to AncCAR-P identified by both algorithms
AncCAR-P30/ AncCAR-A	**107**	**17**	**15.9**
AncCAR-P30/ AncCAR-F	**117**	**26**	**22.2**

Supplementary figure 5 – AncCARs derived from the most likely sequence output from different algorithms incorporate different variation than an AncCAR from PAML's posterior probability table with residues incorporated at a 30% probability cut-off.

Data shows that AncCAR algorithms sample ancestral space in a different manner than the variation derived from variation from the table of posterior probabilities. AncCAR-P30 was generated from the table of posterior probabilities output by PAML using a bespoke python script. Sequences were aligned with MUSCLE in Geneious v. 10. Sites containing gaps were omitted from the analysis. Variation was identified by eye and transposed into Microsoft Excel. **A)** An identity barcode comparing residue conservation at every position in AncCAR-A, AncCAR-F and AncCAR-P30 when compared to AncCAR-P. Coloured lines denote a site with varying amino acid identity to the reference. **B)** Bar chart displaying instances of variation at each residue when comparing AncCAR-P to either AncCAR-A and AncCAR-P30, or AncCAR-F and AncCAR-P30. Data shows that very few positions have equivalent variation derived between algorithmic methods and the posterior probability table of PAML. Vertical lines denote the number of residues that vary at each position in each comparison dataset (min 0, max 2) compared to AncCAR-P.

Supplementary Figure 6

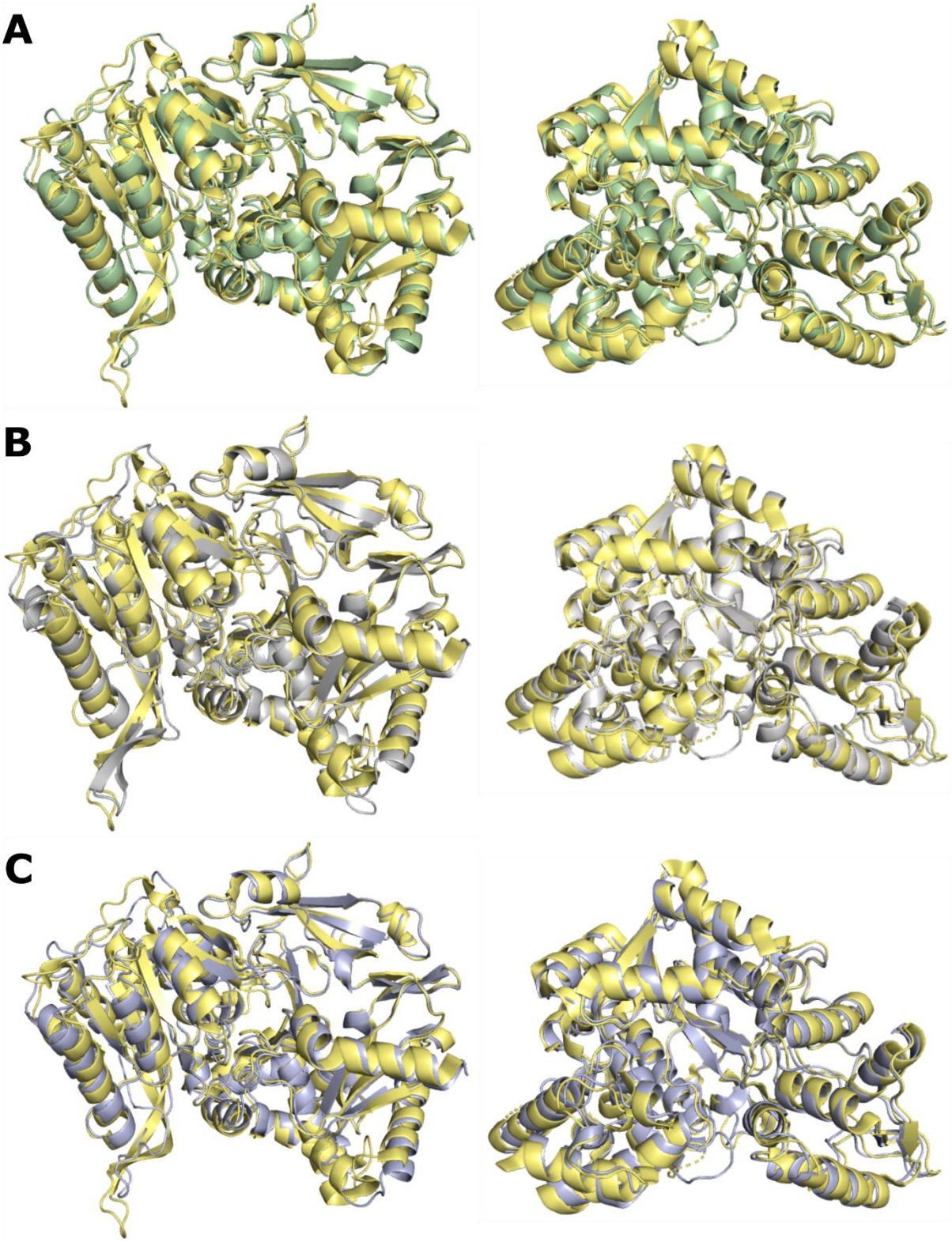

Supplementary figure 6 – Models of AncCARs superimposed on extant structures 5MST/5MSP

Models of the AncCAR adenylation domains (left) and reductase domains (right) superimposed on extant CAR structures 5MST and 5MSO respectively. Structures: Yellow – 5MST; Green - AncCAR-A (**A**); Orange – AncCAR-F (**B**); Blue: AncCAR-PA (**C**) Equivalent images for AncCAR-PF are given in Figure 1C/D. Images produced using PyMOL.

Supplementary figure 7

A

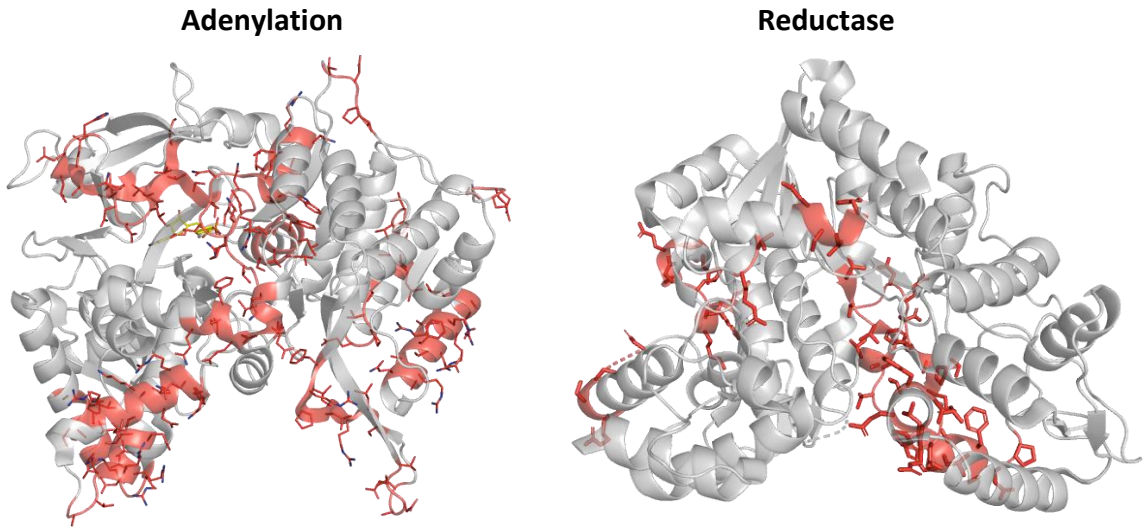

B

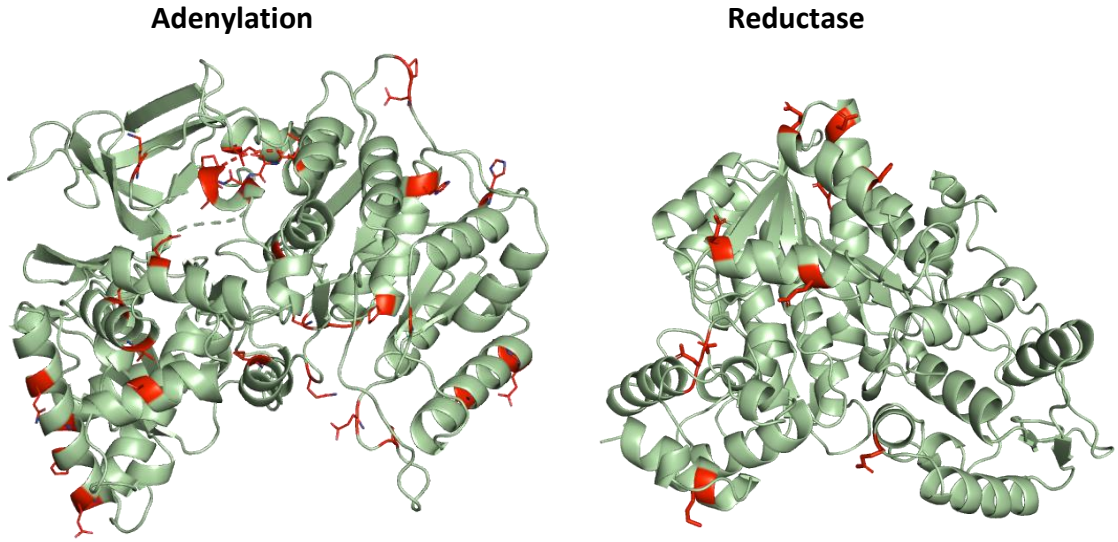

Supplementary figure 7 – Regions of significant sequence variation between AncCARs and 5MST/5MSP

Considerably more sequence variation exists in the adenylation domain of AncCARs compared to the reductase domain. Most this variation occurs on the protein surface on loops. **A)** Sequence alignment between AncCARs and sequences of crystal structures of CAR adenylation domain (5MST) and reductase domain (5MSP) was made in MUSCLE, within the Geneious software suite. Regions of considerable variation were highlighted on the WT crystal structures in red. This is not representative of every change, but regions with multiple changes in sequence. **B)** Sequence alignment between AncCARs was made in MUSCLE within Geneious software suite. Any regions of variation between enzymes (red) were highlighted on the modelled adenylation and reductase domains of AncCAR-A (green). Images were rendered in PyMOL v. 2.0.

Supplementary Figure 8

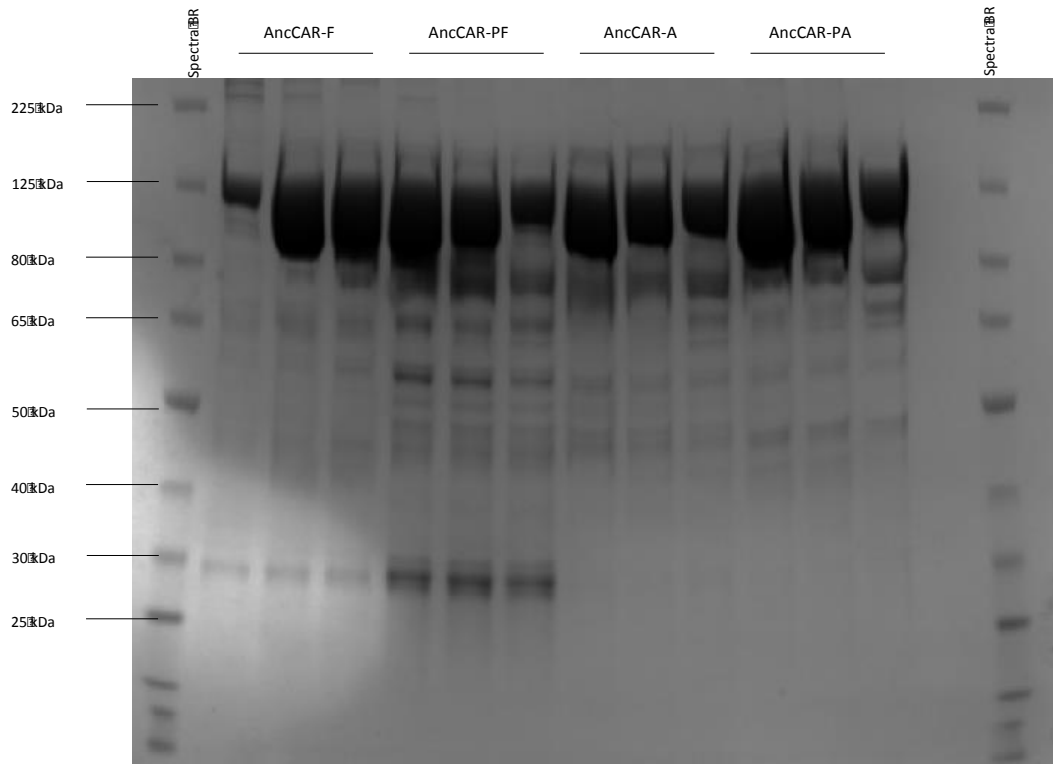

Supplementary Figure 8 – All CAR enzymes are soluble
4-20% acrylamide SDS-PAGE gel of three fractions from the largest peaks following CAR purification by nickel-affinity followed by size exclusion chromatography. All four AncCAR proteins are soluble, producing large volumes of protein (all AncCARs are approximately 128 kDa in size). Typically, per liter bacterial culture, between 3 and 7 mg enzyme could be extracted.

Supplementary Figure 9

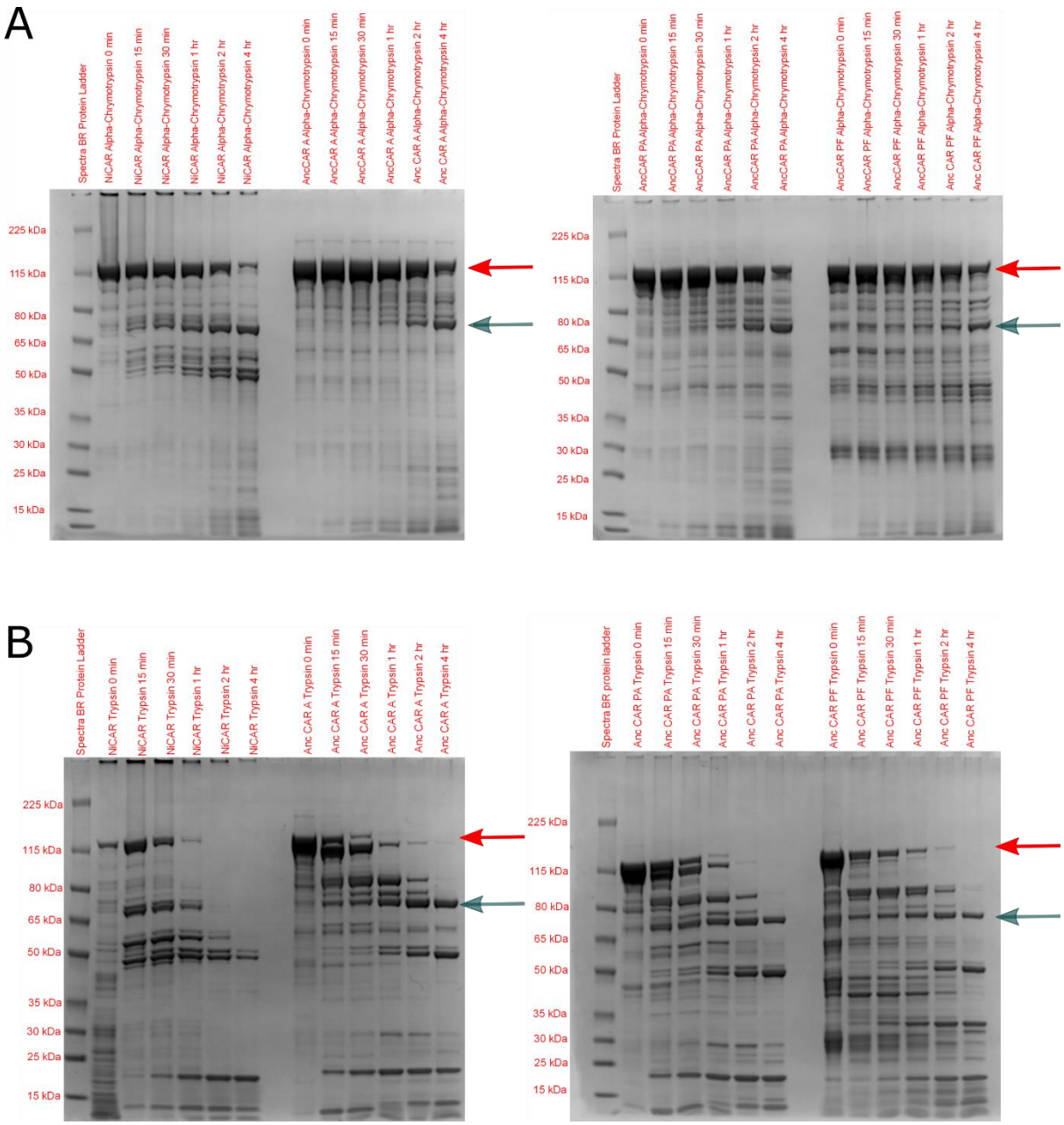

Supplementary Figure 9 – AncCARs are less protease sensitive than ExCARs.
Limited proteolysis was performed on the CAR from *Nocardia iowensis*, AncCAR-A, AncCAR-PA and AncCAR-PF. In each case, chymotrypsin (top) or trypsin (lower) was added at 1 μg/mL to a sample of protein at 1 mg/mL. The proteins were incubated at 37 °C, samples taken at various points and quenched by boiling in SDS-PAGE sample buffer. In both cases, the progression of the proteolysis from the whole protein (red arrow) to separate domains (A domain indicated by the teal arrow) proceeds faster for NiCAR than AncCARs. With chymotrypsin, whole NiCAR is almost entirely lost by 4 hr, whilst AncCARs have considerable amounts intact. With trypsin, all proteins have at least one nick by 1 hr; the A domain of NiCAR has also received at least one nick by 4 hr, whilst it is largely intact for the AncCARs. These results show that AncCARs are less sensitive to these proteases than NiCAR.

Supplementary Figure 10

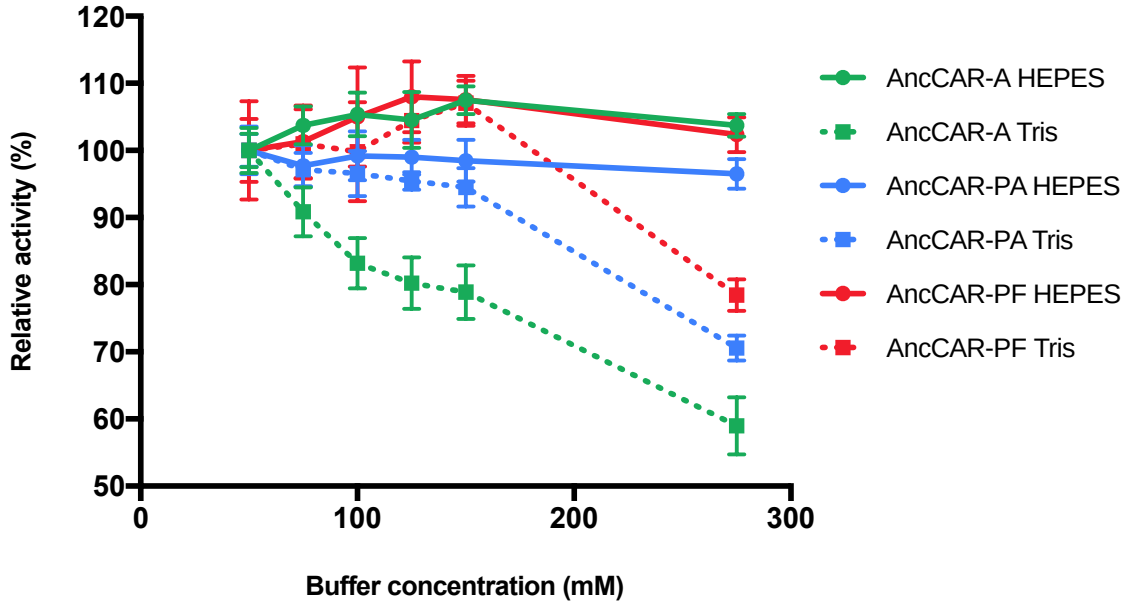

Supplementary Figure 10 – Tris inhibits AncCAR Enzymes
To observe kinetics for AncCARs, high concentrations of substrate may be required, requiring increased concentrations of a buffer system to ensure pH deviations do not affect the rate of NADPH degradation. AncCAR's relative rate (based on the lowest concentration of DMSO tested) of NADPH reduction in the presence of (*E*)-3-phenylprop-2-enoic acid, and increasing concentrations of buffer was tested. At high concentrations of Tris (dotted lines; above 50 mM) AncCAR-A (squares) and AncCAR-PA (inverted triangles) are inhibited. At 275 mM Tris, AncCAR-PF is also inhibited (open circles). However, HEPES (solid lines; circles, triangles and diamonds respectively) shows no apparent inhibitory effects for all AncCARs. Data were visualized in Graphpad Prism v. 7

Supplementary Figure 11

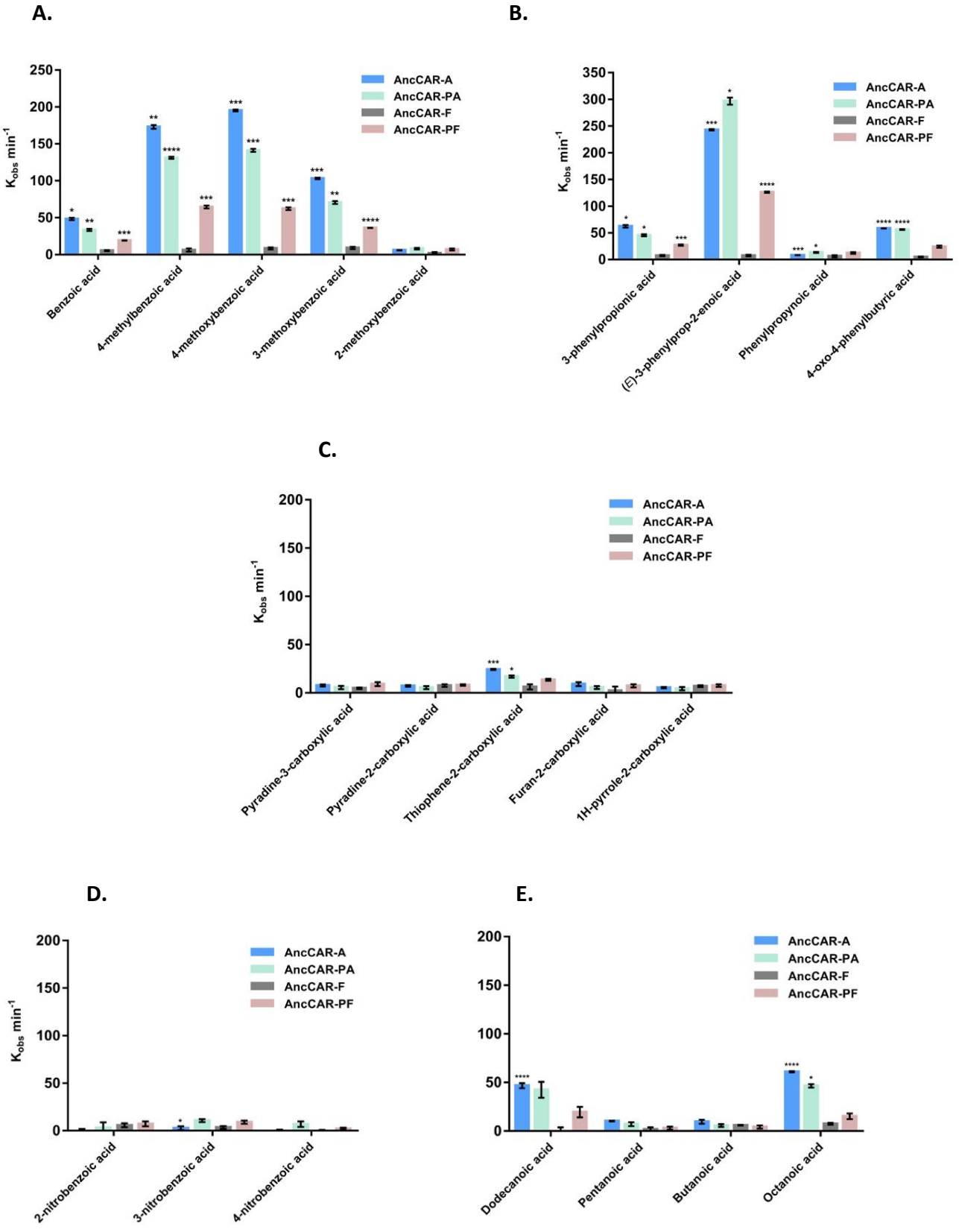

Supplementary Figure 11. **AncCARs have equivalent substrate ranges**
Turnover of NADPH by AncCARs was measured with 24 unique carboxylic acids. Bar charts shows activity on canonical acid substrates at 5 mM. **A)** Benzoic acid its derivatives, **B)** carboxylic acids with a conjugated carboxyl group, **C)** carboxylic acids with substitutions into the aromatic ring, **D)** carboxylic acids with nitro groups, **E)** fatty acids. Each substrate was tested in triplicate, and error bars represent standard error. Asterisks represent degrees of significance from *t*-test of triplicate verses all controls (* = $0.0001 < P \leq 0.001$; ** $0.00001 < P \leq 0.0001$; *** = $0.000001 < P \leq 0.00001$; **** = $P \leq 0.000001$). Substrates were taken forward for kinetic analysis when two or more of the ancestral enzymes showed significant activity. Substrates taken forward for kinetic analysis were: benzoic acid, 4-methylbenzoic acid, 4-methoxybenzoic acid, 3-methoxybenzoic acid, 3-phenylpropionic acid, (*E*)-3-phenylprop-2-enoic acid, phenylpropyonic acid, 4-oxo-4-phenylbutyric acid, thiophene-2-carboxylic acid and octanoic acid. Data were visualized in Graphpad Prism v. 7.

Supplementary Figure 12

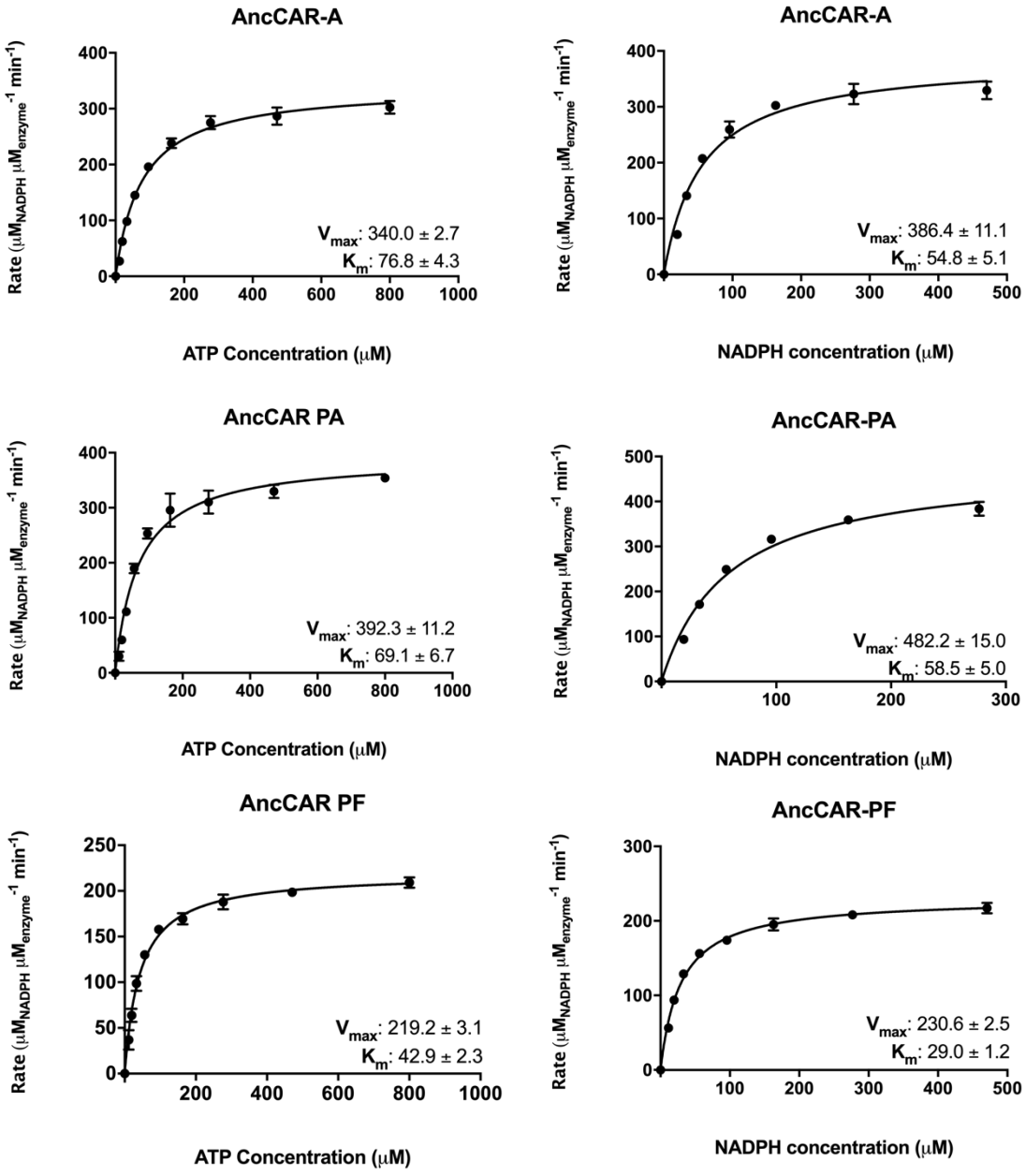

Supplementary figure 12 - AncCAR Kinetics in ATP and NADPH

AncCAR kinetics for NADPH and ATP were obtained from NADPH turnover on (*E*)-3-phenylprop-2-enoic acid. Data were fit to the Michaelis-Menten equation in Graphpad Prism v.7. For NADPH kinetics, measuring rate from the signal producing component of the assay dramatically increases the signal:noise ratio at the low end, and measurable absorbance dips below background within ~3 kinetic cycles. As a result, low end values were also omitted from the curves to avoid a skewing effect. Where the reaction ran to completion fast enough for time between replicate pipetting to be a rate-affecting factor, results were time-adjusted by measuring out 20 second intervals between starting each replicate and adjusting analysis accordingly $V_{max} = k_{cat}$. Units for V_{max}: μM μM^{-1} min^{-1}. Units for K_M: μM.

Supplementary Figure 13

A

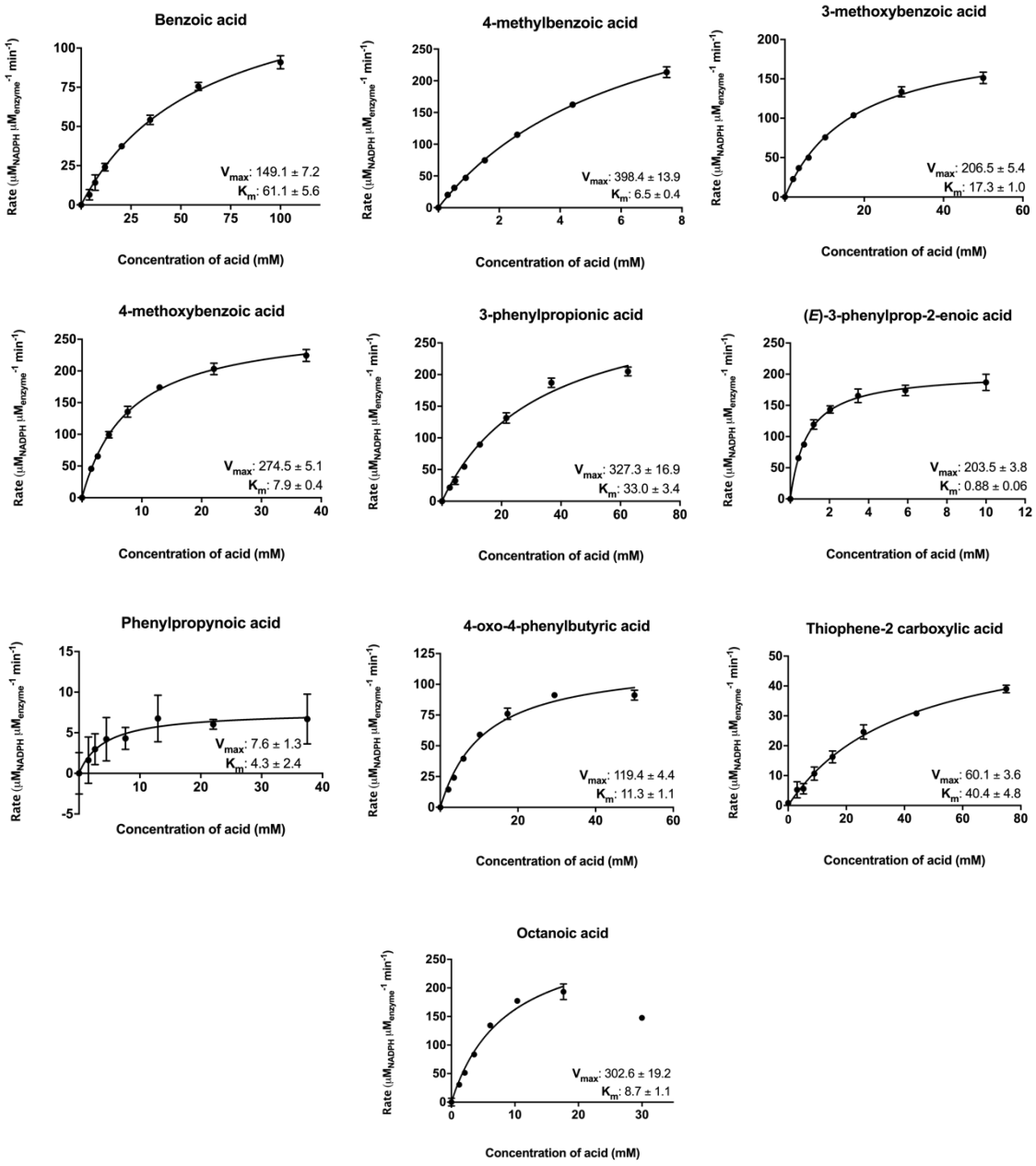

PA

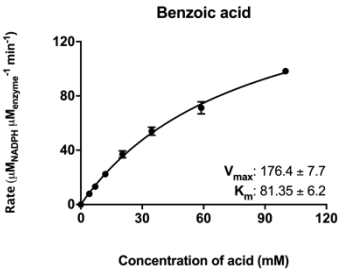
Benzoic acid
Rate (μM$_{NADPH}$ μM$_{enzyme}^{-1}$ min^{-1})
V$_{max}$: 176.4 ± 7.7
K$_m$: 81.35 ± 6.2
Concentration of acid (mM)

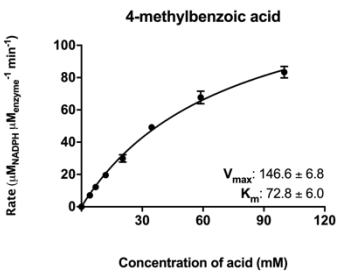
4-methylbenzoic acid
Rate (μM$_{NADPH}$ μM$_{enzyme}^{-1}$ min^{-1})
V$_{max}$: 146.6 ± 6.8
K$_m$: 72.8 ± 6.0
Concentration of acid (mM)

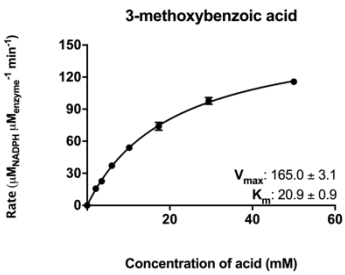
3-methoxybenzoic acid
Rate (μM$_{NADPH}$ μM$_{enzyme}^{-1}$ min^{-1})
V$_{max}$: 165.0 ± 3.1
K$_m$: 20.9 ± 0.9
Concentration of acid (mM)

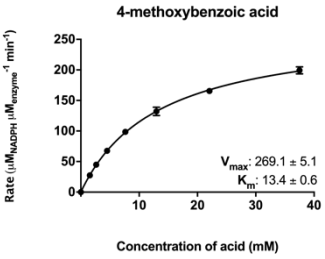
4-methoxybenzoic acid
Rate (μM$_{NADPH}$ μM$_{enzyme}^{-1}$ min^{-1})
V$_{max}$: 269.1 ± 5.1
K$_m$: 13.4 ± 0.6
Concentration of acid (mM)

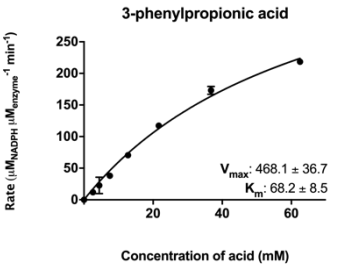
3-phenylpropionic acid
Rate (μM$_{NADPH}$ μM$_{enzyme}^{-1}$ min^{-1})
V$_{max}$: 468.1 ± 36.7
K$_m$: 68.2 ± 8.5
Concentration of acid (mM)

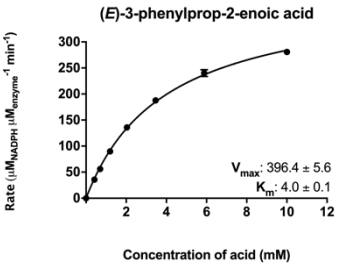
(E)-3-phenylprop-2-enoic acid
Rate (μM$_{NADPH}$ μM$_{enzyme}^{-1}$ min^{-1})
V$_{max}$: 396.4 ± 5.6
K$_m$: 4.0 ± 0.1
Concentration of acid (mM)

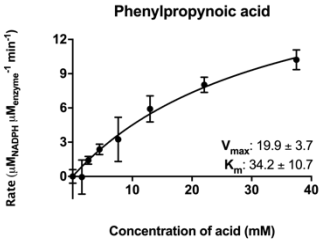
Phenylpropynoic acid
Rate (μM$_{NADPH}$ μM$_{enzyme}^{-1}$ min^{-1})
V$_{max}$: 19.9 ± 3.7
K$_m$: 34.2 ± 10.7
Concentration of acid (mM)

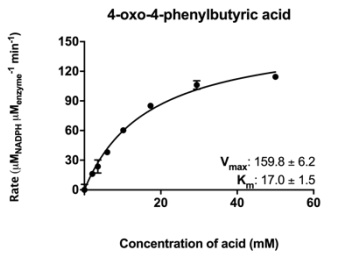
4-oxo-4-phenylbutyric acid
Rate (μM$_{NADPH}$ μM$_{enzyme}^{-1}$ min^{-1})
V$_{max}$: 159.8 ± 6.2
K$_m$: 17.0 ± 1.5
Concentration of acid (mM)

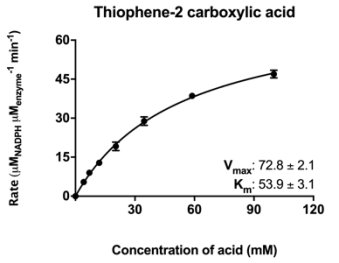
Thiophene-2 carboxylic acid
Rate (μM$_{NADPH}$ μM$_{enzyme}^{-1}$ min^{-1})
V$_{max}$: 72.8 ± 2.1
K$_m$: 53.9 ± 3.1
Concentration of acid (mM)

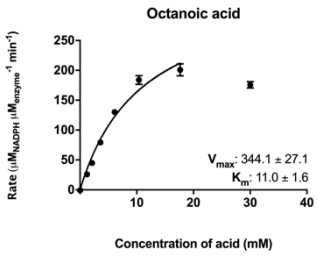
Octanoic acid
Rate (μM$_{NADPH}$ μM$_{enzyme}^{-1}$ min^{-1})
V$_{max}$: 344.1 ± 27.1
K$_m$: 11.0 ± 1.6
Concentration of acid (mM)

PF

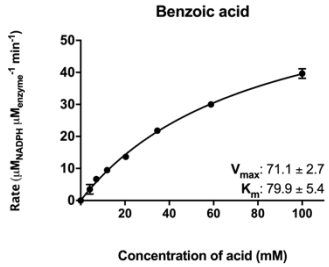
Benzoic acid
Rate (μM$_{NADPH}$ μM$_{enzyme}^{-1}$ min^{-1})
V$_{max}$: 71.1 ± 2.7
K$_m$: 79.9 ± 5.4
Concentration of acid (mM)

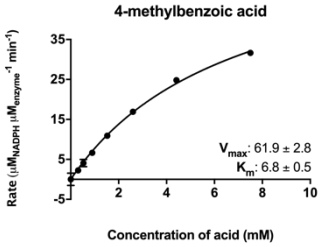
4-methylbenzoic acid
Rate (μM$_{NADPH}$ μM$_{enzyme}^{-1}$ min^{-1})
V$_{max}$: 61.9 ± 2.8
K$_m$: 6.8 ± 0.5
Concentration of acid (mM)

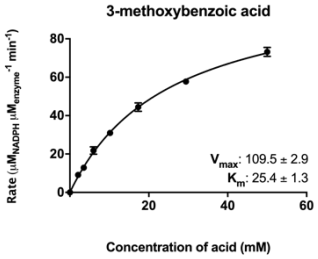
3-methoxybenzoic acid
Rate (μM$_{NADPH}$ μM$_{enzyme}^{-1}$ min^{-1})
V$_{max}$: 109.5 ± 2.9
K$_m$: 25.4 ± 1.3
Concentration of acid (mM)

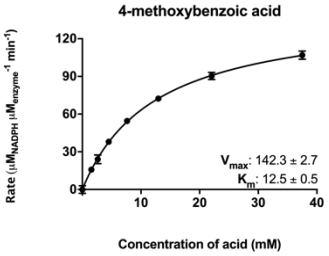
4-methoxybenzoic acid
Rate (μM$_{NADPH}$ μM$_{enzyme}^{-1}$ min^{-1})
V$_{max}$: 142.3 ± 2.7
K$_m$: 12.5 ± 0.5
Concentration of acid (mM)

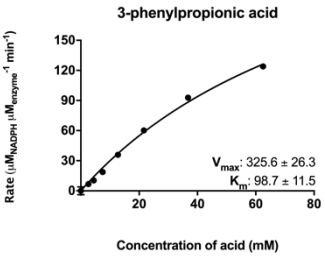
3-phenylpropionic acid
Rate (μM$_{NADPH}$ μM$_{enzyme}^{-1}$ min^{-1})
V$_{max}$: 325.6 ± 26.3
K$_m$: 98.7 ± 11.5
Concentration of acid (mM)

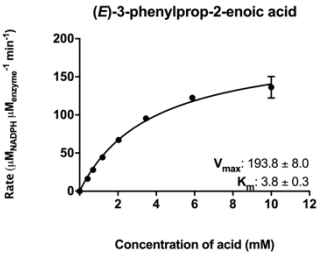
(E)-3-phenylprop-2-enoic acid
Rate (μM$_{NADPH}$ μM$_{enzyme}^{-1}$ min^{-1})
V$_{max}$: 193.8 ± 8.0
K$_m$: 3.8 ± 0.3
Concentration of acid (mM)

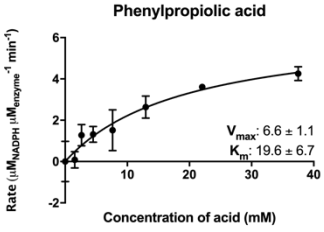
Phenylpropiolic acid
Rate (μM$_{NADPH}$ μM$_{enzyme}^{-1}$ min^{-1})
V$_{max}$: 6.6 ± 1.1
K$_m$: 19.6 ± 6.7
Concentration of acid (mM)

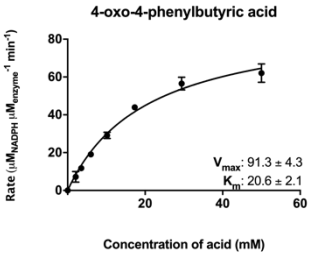
4-oxo-4-phenylbutyric acid
Rate (μM$_{NADPH}$ μM$_{enzyme}^{-1}$ min^{-1})
V$_{max}$: 91.3 ± 4.3
K$_m$: 20.6 ± 2.1
Concentration of acid (mM)

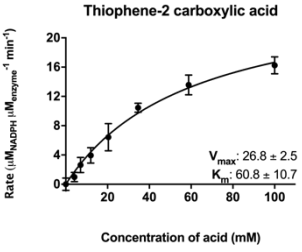
Thiophene-2 carboxylic acid
Rate (μM$_{NADPH}$ μM$_{enzyme}^{-1}$ min^{-1})
V$_{max}$: 26.8 ± 2.5
K$_m$: 60.8 ± 10.7
Concentration of acid (mM)

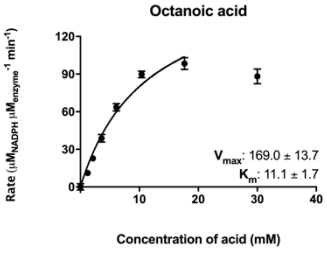
Octanoic acid
Rate (μM$_{NADPH}$ μM$_{enzyme}^{-1}$ min^{-1})
V$_{max}$: 169.0 ± 13.7
K$_m$: 11.1 ± 1.7
Concentration of acid (mM)

B		Benzoic acid	4-methylbenzoic acid	4-methoxybenzoic acid	3-methoxybenzoic acid	3-phenylpropionic acid
A	k_{cat} (min^{-1})	149.1 ± 7.1	398.4 ± 13.9	274.5 ± 5.1	206.5 ± 5.4	327.3 ± 16.9
	K_M (mM)	61.2 ± 5.6	6.5 ± 0.4	7.9 ± 0.4	17.3 ± 1.0	33.0 ± 3.4
	k_{cat}/K_M (min^{-1} mM^{-1})	2.4 ± 0.3	61.3 ± 4.3	34.7 ± 1.9	11.9 ± 0.8	9.9 ± 1.1
PA	k_{cat} (min^{-1})	176.4 ± 7.7	146.6 ± 6.8	269.1 ± 5.1	165 ± 3.1	468.1 ± 36.7
	K_M (mM)	81.4 ± 6.2	5.5 ± 0.5	13.4 ± 0.6	20.9 ± 0.9	68.2 ± 8.5
	k_{cat}/K_M (min^{-1} mM^{-1})	2.2 ± 0.2	26.7 ± 2.7	20.1 ± 1.0	7.9 ± 0.4	6.9 ± 1.0
PF	k_{cat} (min^{-1})	71.8 ± 2.7	61.9 ± 2.8	142 ± 2.7	109.5 ± 2.9	325.6 ± 26.3
	K_M (mM)	79.9 ± 5.4	7.0 ± 0.5	12.5 ± 0.6	25.4 ± 1.3	98.7 ± 11.5
	k_{cat}/K_M (min^{-1} mM^{-1})	0.9 ± 0.1	8.8 ± 0.7	11.4 ± 0.6	4.3 ± 0.2	3.3 ± 0.5

		(E)-3-phenylprop-2-enoic acid	Phenylpropyonic acid	4-oxo-4-phenylbutyric acid	2-thiophene carboxylic acid	Octanoic acid
A	K_{cat} (min^{-1})	203.5 ± 3.8	7.6 ± 1.3	119.4 ± 4.4	60.1 ± 3.6	302.6 ± 19.2
	K_M (mM)	0.9 ± 0.06	4.3 ± 2.4	11.3 ± 1.1	40.4 ± 4.8	8.7 ± 1.1
	K_{cat}/K_M (min^{-1} mM^{-1})	226.1 ± 15.7	1.8 ± 2.4	10.6 ± 1.1	1.5 ± 0.2	34.8 ± 4.9
PA	K_{cat} (min^{-1})	396.4 ± 5.6	19.9 ± 3.7	159.8 ± 6.2	72.8 ± 2.1	344.1 ± 27.1
	K_M (mM)	4.0 ± 0.1	34.2 ± 10.8	17.0 ± 1.5	53.9 ± 3.1	11.0 ± 1.6
	K_{cat}/K_M (min^{-1} mM^{-1})	99.1 ± 2.8	0.6 ± 0.2	9.4 ± 0.9	1.4 ± 0.1	31.3 ± 5.2
PF	K_{cat} (min^{-1})	193.8 ± 8.0	6.6 ± 1.1	91.3 ± 4.3	26.8 ± 2.5	169.0 ± 13.7
	K_M (mM)	3.8 ± 0.3	19.6 ± 6.7	20.6 ± 2.1	60.8 ± 10.7	11.1 ± 1.7
	K_{cat}/K_M (min^{-1} mM^{-1})	51.0 ± 4.5	0.34 ± 0.1	4.4 ± 0.5	0.4 ± 0.1	15.2 ± 4.2

Supplementary figure 13

AncCAR kinetics were calculated from NADPH turnover by AncCARs in the presence of the 10 substrates exhibiting significant activity in supplementary figure 9. 10 µg enzyme were used to improve resolution of 4-methylbenzoic acid and phenylpropiolic acid. Kinetics were determined using an 8 point, 1.7x dilution series of acid from near saturation in 200 mM HEPES, with concentrations starting at 800 mM. Each concentration was investigated in triplicate. Data were fitted to the Michaelis-Menten equation in Graphpad Prism v. 7. V_{max} = k_{cat}. Units for V_{max}: µM µM^{-1} min^{-1}. Units for K_M: µM.

Supplementary Figure 14

A

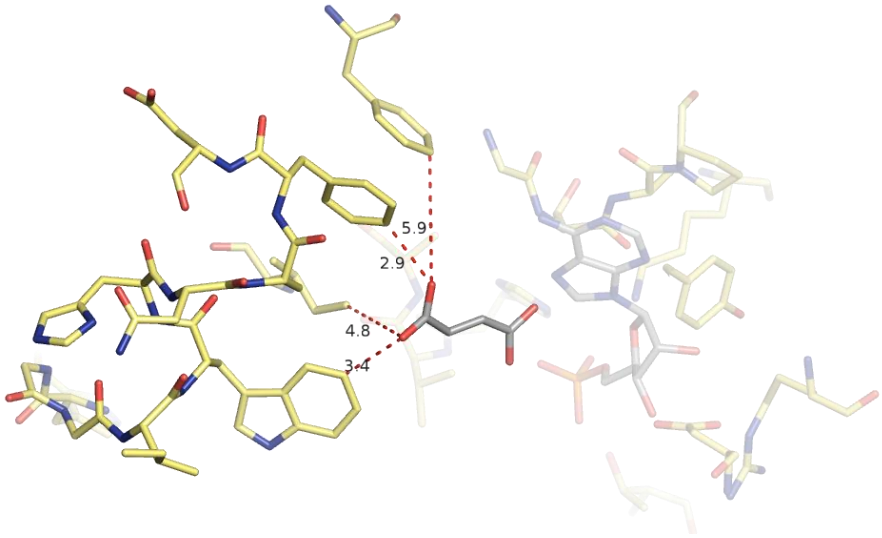

B

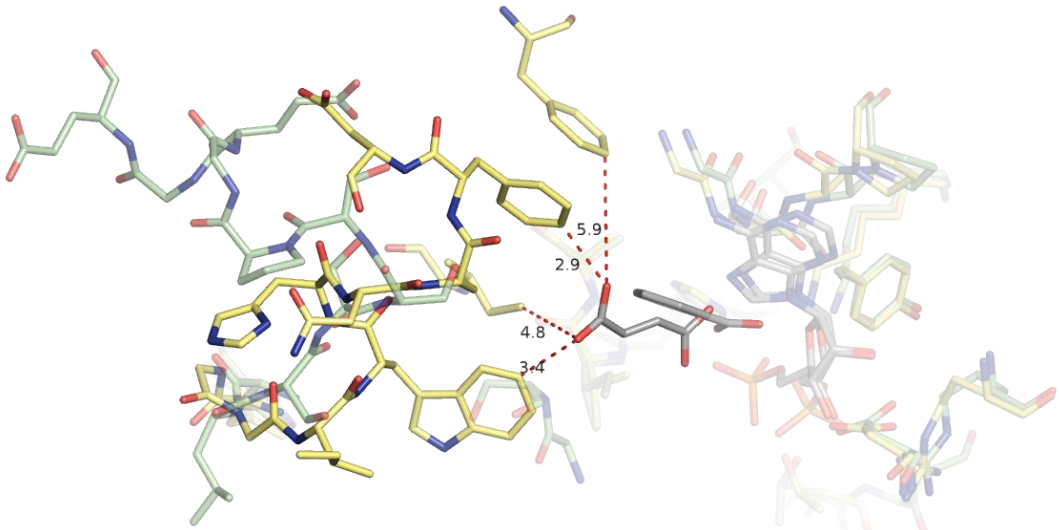

C

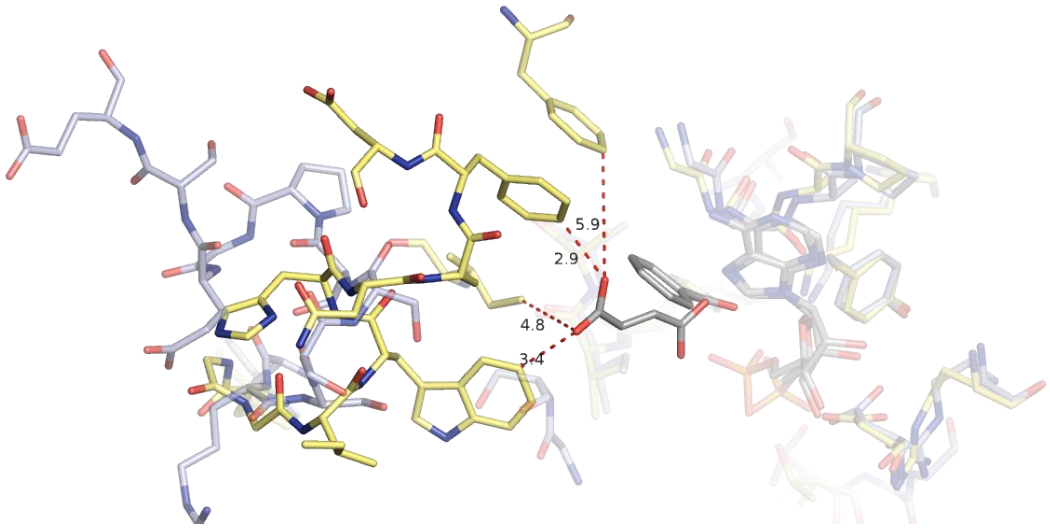

D

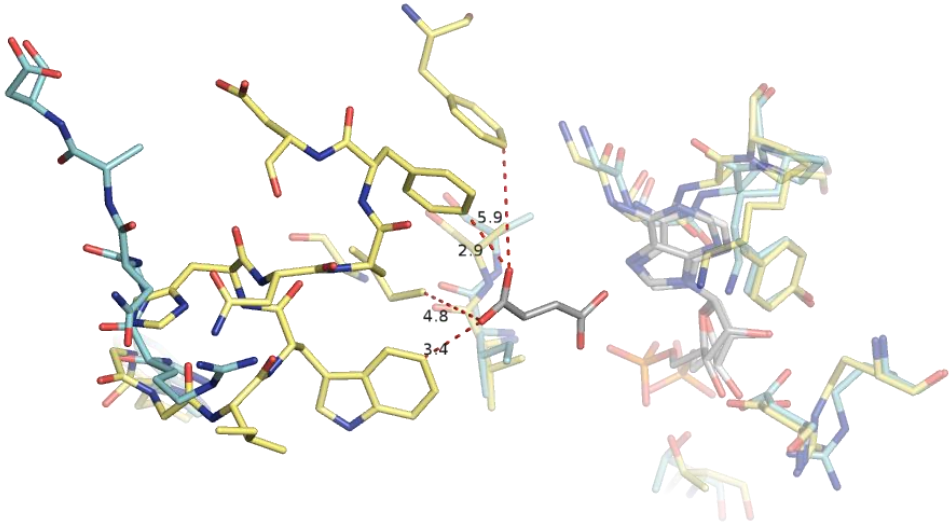

Supplementary Figure 14
Close-up analysis of models of the highly variable loop in the adenylation domain of AncCARs compared to 5MST (286-302) show that AncCAR-PF (Figure 2E) and AncCAR-F do not form potentially stabilizing interactions with the substrate in this region. **A)** Close-up image of 5MST active site. **B)** Modelled structure of AncCAR-A overlaid onto 5MST **C)** Modelled structure of AncCAR-PA overlaid onto 5MST. **D)** Modelled structure of AncCAR-F overlaid onto 5MST. Models were rendered in PyMOL v. 2.0.

Supplementary Figure 15

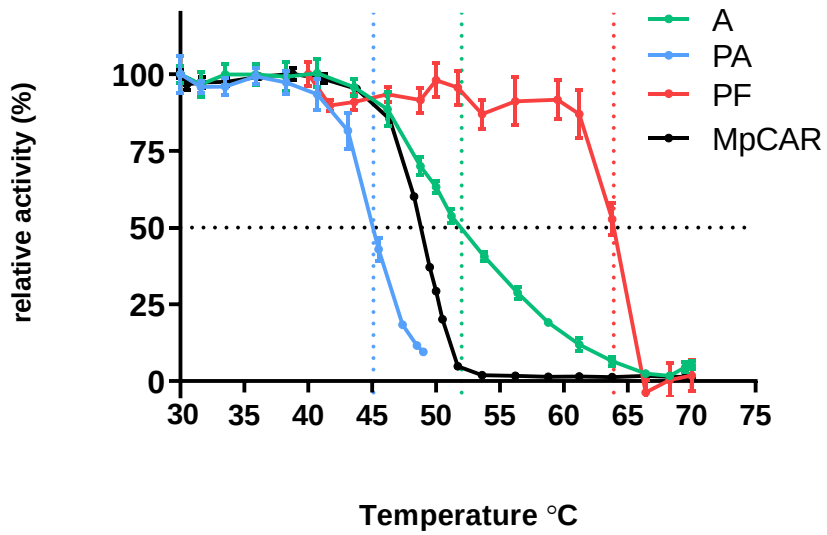

Supplementary figure 15

CARs were incubated at temperatures from 30 °C to 70 °C for 30 min in 10 mM HEPES and 500 mM NaCl. Each point represents the rate of NADPH oxidation in 5 mM (*E*)-3-phenylprop-2-enoic acid relative to the rate of NADPH oxidation in 5 mM (*E*)-3-phenylprop-2-enoic acid at 30 °C. Each point represents a single triplicate, with error bars representing the standard error. 50% activity was lost at 45 °C, 52 °C, 64 °C and 49 °C for AncCAR-PA, AncCAR-A, AncCAR-PF and MpCAR respectively. Data were visualized in Graphpad Prism v. 7.

Supplementary Figure 16

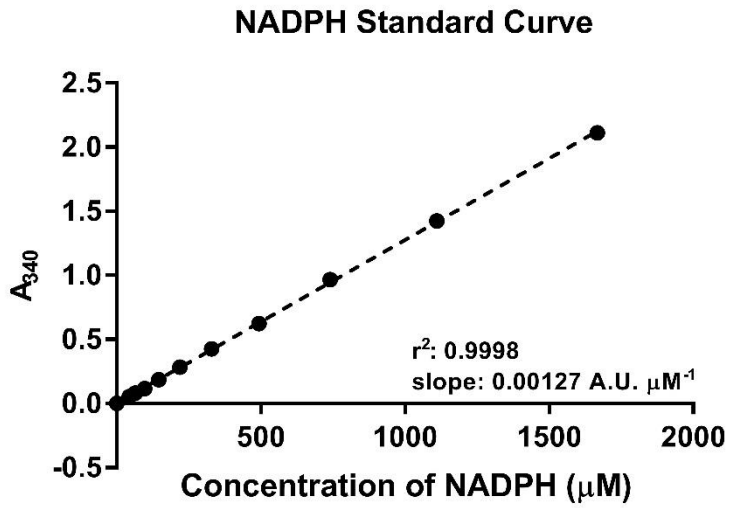

Supplementary figure 16: NADPH standard curve
An NADPH standard curve was constructed for conversion of raw assay data into substrate turnover. The curve was created by titration of NADPH from 1,700 μM in a 1.5x dilution series in standard reaction buffer in triplicate. Absorbance of the solution was measured at 340 nm. Error bars are occluded by the data points. Data were visualized in Graphpad Prism v. 7.

Supplementary Table 1

	Adenylation		Reductase	
	5MST	5MSD	5MSP	5MSO
AncCAR-A	0.987	0.372	0.898	0.944
AncCAR-PA	1.011	0.444	1.061	1.115
AncCAR-PF	1.276	0.872		
AncCAR-F	1.131	0.747	1.21	0.804
5MST		0.964		
5MSD	0.964			
5MSP				0.701
5MSO			0.701	

***t*-test A→R**	$p = 0.13$

Supplementary Table 1– Root mean squared values of alpha carbon atom displacement in AncCAR protein models - showing good fit of data.

Rmsd values of alpha carbon displacement between modelled AncCAR structures were calculated in PyMOL v 2.0. Displacement values give confidence that the protein modelling is represents a good homology model to the extant structures. *t*-test shows that there is not significantly more displacement between the adenylation and reductase domains, showing that the modelled AncCAR domains are modelled to equal confidence. Structures 5MST and 5MSP are from CAR from *Segniliparus rugosus* (representative of the outgroup in Supplementary Figure 2). Structure 5MSD and 5MSO are from CARs from *Nocardia iowensis* and *Mycobacterium marinum*.

Supplementary Table 2

	Solvent		A	PA	PF	NiCAR	MpCAR	St. Dev.
**Polar aprotic**	Acetone	A_{50}	14.3	20.4	17.9	17.3	14.0	3.1
		S_{10}	64.4	76.3	68.8	73.9	62.1	6.0
	Acetonitrile	A_{50}	11.8	16.7	20.2	8.4	9.8	4.2
		S_{10}	57.3	78.3	93.4	41.6	49.3	18.1
	**DMSO**	**A_{50}**	**23.9**	**23.7**	**24.3**	**>25**	**19.5**	**0.3**
		**S_{10}**	**88.9**	**86.5**	**92.4**	**67.4**	**73.7**	**3.0**
**Polar protic**	Ethanol	A_{50}	10.0	14.9	17.7	8.2	21.9	3.9
		S_{10}	50.1	66.5	72.6	41.5	81.2	11.6
	Isopropanol	A_{50}	7.3	11.7	11.8	9.0	15.9	2.6
		S_{10}	36.3	56.8	56.6	45.3	73.0	11.8
	**Methanol**	**A_{50}**	**14.1**	**11.7**	**25.6**	**8.6**	**22.0**	**7.5**
		**S_{10}**	**63.3**	**56.8**	**78.6**	**43.6**	**82.1**	**11.2**

Supplementary table 2 - AncCAR tolerance to various solvents
AncCARs activity on 5 mM (*E*)-3-phenylprop-2-enoic acid was assessed in the presence of aprotic and protic solvents by solvent titration from 25% (v/v). Inhibition curves were fit to a second order polynomial in GraphPad Prism v. 7, from which A_{50} (% solvent at which 50% of activity is lost) and S_{10} (%activity at 10% solvent relative to 0% solvent) were calculated. Emboldened text represent data for Figures 4A and 4B.

References

1. Gahloth, D. et al. Structures of carboxylic acid reductase reveal domain dynamics underlying catalysis. *Nature Chemical Biology* **13**, 975-981 (2017).
2. Finnigan, W. et al. Characterization of Carboxylic Acid Reductases as Enzymes in the Toolbox for Synthetic Chemistry. *ChemCatChem* **9**, 1005–1017 (2017).
3. Ronquist, F. et al. MrBayes 3.2: Efficient Bayesian Phylogenetic Inference and Model Choice Across a Large Model Space. *Systematic Biology* **61**, 539–542 (2012).
4. Whelan, S. & Goldman, N. A general empirical model of protein evolution derived from multiple protein families using a maximum-likelihood approach. Molecular Biology & Evolution **18**, 691–699 (2001).
5. Li, W.Z. et al. The EMBL-EBI bioinformatics web and programmatic tools framework. *Nucleic Acids Research* **43**, W580-W584 (2015).
