# Supplementary material for: Highly thermostable carboxylic acid reductases generated by ancestral sequence reconstruction
Source: Commun Biol. 2019 Nov 22;2:429. doi: 10.1038/s42003-019-0677-y (PMC6874671; doi:10.1038/s42003-019-0677-y)
Supplement: Supplementary file 2 — Reporting Summary [file 42003_2019_677_MOESM2_ESM.pdf]

nature research

Corresponding author(s): Nicholas Harmer

Last updated by author(s): Aug 20, 2019

# Reporting Summary

Nature Research wishes to improve the reproducibility of the work that we publish. This form provides structure for consistency and transparency in reporting. For further information on Nature Research policies, see Authors & Referees and the Editorial Policy Checklist.

## Statistics

For all statistical analyses, confirm that the following items are present in the figure legend, table legend, main text, or Methods section.

| n/a | Confirmed | |
|---|---|---|
| ☐ | ☒ | The exact sample size (*n*) for each experimental group/condition, given as a discrete number and unit of measurement |
| ☐ | ☒ | A statement on whether measurements were taken from distinct samples or whether the same sample was measured repeatedly |
| ☐ | ☒ | The statistical test(s) used AND whether they are one- or two-sided<br>*Only common tests should be described solely by name; describe more complex techniques in the Methods section.* |
| ☒ | ☐ | A description of all covariates tested |
| ☒ | ☐ | A description of any assumptions or corrections, such as tests of normality and adjustment for multiple comparisons |
| ☒ | ☐ | A full description of the statistical parameters including central tendency (e.g. means) or other basic estimates (e.g. regression coefficient) AND variation (e.g. standard deviation) or associated estimates of uncertainty (e.g. confidence intervals) |
| ☐ | ☒ | For null hypothesis testing, the test statistic (e.g. *F*, *t*, *r*) with confidence intervals, effect sizes, degrees of freedom and *P* value noted<br>*Give P values as exact values whenever suitable.* |
| ☒ | ☐ | For Bayesian analysis, information on the choice of priors and Markov chain Monte Carlo settings |
| ☒ | ☐ | For hierarchical and complex designs, identification of the appropriate level for tests and full reporting of outcomes |
| ☒ | ☐ | Estimates of effect sizes (e.g. Cohen's *d*, Pearson's *r*), indicating how they were calculated |

*Our web collection on statistics for biologists contains articles on many of the points above.*

## Software and code

Policy information about availability of computer code

| | |
|---|---|
| Data collection | Ancestral reconstruction was performed using Geneious v. 10.0.2, ProtTest version 3.4, GBlocks , MrBayes v. 3.2.6, PAML v4.9, FastML (server at http://fastml.tau.ac.il/) and Ancescon (server at http://prodata.swmed.edu/ancescon/ancescon.php). Protein modelling was performed using YASARA v.17.8.15. |
| Data analysis | Data were analysed using GraphPad v. 8.0; Protein Thermal Shift software (Applied Biosystems) v.1.3; and PyMOL v. 2.0. |

For manuscripts utilizing custom algorithms or software that are central to the research but not yet described in published literature, software must be made available to editors/reviewers. We strongly encourage code deposition in a community repository (e.g. GitHub). See the Nature Research guidelines for submitting code & software for further information.

## Data

Policy information about availability of data

All manuscripts must include a data availability statement. This statement should provide the following information, where applicable:

- Accession codes, unique identifiers, or web links for publicly available datasets
- A list of figures that have associated raw data
- A description of any restrictions on data availability

The datasets generated during and/or analysed during the current study are available from the corresponding author on reasonable request. Data are available for Figures 1-4 and Tables 1-2.

# Field-specific reporting

Please select the one below that is the best fit for your research. If you are not sure, read the appropriate sections before making your selection.

☒ Life sciences ☐ Behavioural & social sciences ☐ Ecological, evolutionary & environmental sciences

For a reference copy of the document with all sections, see nature.com/documents/nr-reporting-summary-flat.pdf

# Life sciences study design

All studies must disclose on these points even when the disclosure is negative.

| | |
|---|---|
| Sample size | For determination of the Michaelis-Menten parameters, 24 data points were taken: this is sufficient to fit two parameters well with sufficient degrees of freedom where sample concentrations are selected well.<br>For enzyme activity assays, three experimental replicates were taken. The error for these replicates is low in this system. In most cases, data trends over time were studied, and so results were considered over many data points. |
| Data exclusions | No data were excluded from the study. |
| Replication | All results reported are reflective of multiple experiments undertaken on different days, and using different protein preparations. Each experiment required refinement. The results reported are those from the refined experiments, and these are consistent with the preparatory, optimisation experiments. |
| Randomization | This is not relevant to this study. |
| Blinding | Blinding is not relevant to this study. As an enzyme activity study, the results reflect the effect of a protein against multiple substrates or concentrations of a single substrate. The best comparable data are obtained by collecting all these data together, and getting a liquid handling robot to prepare the samples. |

# Reporting for specific materials, systems and methods

We require information from authors about some types of materials, experimental systems and methods used in many studies. Here, indicate whether each material, system or method listed is relevant to your study. If you are not sure if a list item applies to your research, read the appropriate section before selecting a response.

## Materials & experimental systems

| n/a | Involved in the study | |
|---|---|---|
| ☒ | ☐ | Antibodies |
| ☒ | ☐ | Eukaryotic cell lines |
| ☒ | ☐ | Palaeontology |
| ☒ | ☐ | Animals and other organisms |
| ☒ | ☐ | Human research participants |
| ☒ | ☐ | Clinical data |

## Methods

| n/a | Involved in the study | |
|---|---|---|
| ☒ | ☐ | ChIP-seq |
| ☒ | ☐ | Flow cytometry |
| ☒ | ☐ | MRI-based neuroimaging |
